# Supplementary material for: Nanomedicine-based co-delivery of a calcium channel inhibitor and a small molecule targeting CD47 for lung cancer immunotherapy
Source: Nat Commun. 2023 Nov 11;14:7306. doi: 10.1038/s41467-023-42972-2 (PMC10640620; doi:10.1038/s41467-023-42972-2)
Supplement: Supplementary file 1 — Supplementary Information [file 41467_2023_42972_MOESM1_ESM.pdf]

Supplementary Information

Nanomedicine-based co-delivery of a calcium channel inhibitor and a small molecule targeting CD47 for lung cancer immunotherapy

Yuedong Guo[1,2], Qunqun Bao[3], Ping Hu[1,3]* and Jianlin Shi[1,3]*

[1]State Key Laboratory of High Performance Ceramics and Superfine Microstructures, Shanghai Institute of Ceramics, Chinese Academy of Sciences, Research Unit of Nanocatalytic Medicine in Specific Therapy for Serious Disease, Chinese Academy of Medical Sciences (2021RU012), Shanghai 200050, P. R. China.

[2]Center of Materials Science and Optoelectronics Engineering, University of Chinese Academy of Sciences, Beijing, 100049 P. R. China.

[3]Shanghai Tenth People's Hospital, Shanghai Frontiers Science Center of Nanocatalytic Medicine, School of Medicine, Tongji University, Shanghai 200331, P. R. China.

Corresponding authors: Ping Hu (huping@mail.sic.ac.cn) and Jianlin Shi (jlshi@mail.sic.ac.cn)

Supplementary Tables

Supplementary Table 1. Abbreviation table.

Full name	Abbreviation	Full name	Abbreviation
Layered double hydroxide	LDH	T helper type 1	Th1
Inductively coupled plasma optical emission spectrometry	ICP-OES	1,1'-dioctadecyl-3,3,3',3'-tetramethylindocarbocyanine perchlorate	DiI
Calreticulin	CRT	Indocyanine Green	ICG
Immune checkpoint block	ICB	Regulatory T cells	Treg cells
Natural killer cells	NK cells	Endoplasmic reticulum	ER
Dendritic cells	DCs	Fourier transform infrared spectra	FTIR
Bone marrow-derived macrophages	BMDMs	Polyethylene glycol	PEG
Alveolar macrophages	AMs	X-ray diffraction	XRD
Interstitial macrophages	IMs	Phosphate buffered saline	PBS
Transforming growth factor-β	TGF-β	Median fluorescence intensity	MFI
Programmed cells death protein 1	PD-1	LDH/RRX-001	LR
Programmed cells death protein 1 ligand 1	PD-L1	LDH/TTA-Q6	LT
Bone marrow-derived dendritic cells	BMDCs	Lewis lung carcinoma	LLC
LDH/RRX-001/TTA-Q6	LRT	Propidium Iodide	PI
Tumor associated macrophages	TAMs	4',6-diamidino-2-phenylindole	DAPI
Transmission electron microscopy	TEM	Confocal laser scanning microscopy	CLSM
Selected area electron diffraction	SAED	Antibody dependent cell-mediated cytotoxicity	ADCC
Scanning electron micrograph	SEM	Tumor necrosis factor-α	TNF-α
Energy-dispersive X-ray spectroscopy	EDS	Interleukin-1β	IL-1β
Interleukin-6	IL-6	Interferon gamma	IFN-γ
Carboxyfluorescein Succinimidyl Ester	CFSE		

Supplementary Table 2. The statistic results of Figure 4e showing flow cytometry analysis for the AMs' immunophenotype, tumor cell phagocytosis and killing effect. (MC: metacluster)

Groups	MC 1	MC 2	MC 3	MC 4	MC 5	MC 6	MC 7	MC 8
**Control**	0.76%	9.76%	0.15%	77.59%	0.13%	0.63%	10.93%	0.05%
**LDH**	0.54%	5.55%	0.16%	77.63%	0.14%	1.24%	14.69%	0.05%
**LR**	7.16%	1.25%	0.01%	0.52%	0.11%	2.46%	88.1%	0.37%
**LT**	5.77%	1.07%	0.01%	0.64%	0.16%	2.54%	89.41%	0.4%
**LRT**	6.25%	1.03%	0.02%	0.64%	0.21%	3.22%	88.14%	0.49%

Supplementary Table 3. The percentages of LLC cells in Supplementary Table 2.

Groups	Late apoptosis LLC cells	Live LLC cells
**Control**	7.22%	92.78%
**LDH**	8.87%	91.13%
**LR**	85.14%	14.86%
**LT**	84.36%	15.64%
**LRT**	85.85%	14.15%

Supplementary Table 4. The percentages of AMs in Supplementary Table 2.

Groups	Non-M1 AMs that phagocytized LLC cells	Non-M1 AMs	Dead M1 AMs	Live M1 AMs	M1 AMs that phagocytized LLC cells
**Control**	0.17%	86.84%	0.71%	12.23%	0.06%
**LDH**	0.17%	82.79%	1.32%	15.67%	0.05%
**LR**	0.01%	0.57%	2.69%	96.33%	0.40%
**LT**	0.01%	0.69%	2.73%	96.14%	0.43%
**LRT**	0.02%	0.69%	3.48%	95.28%	0.53%

Supplementary Figures

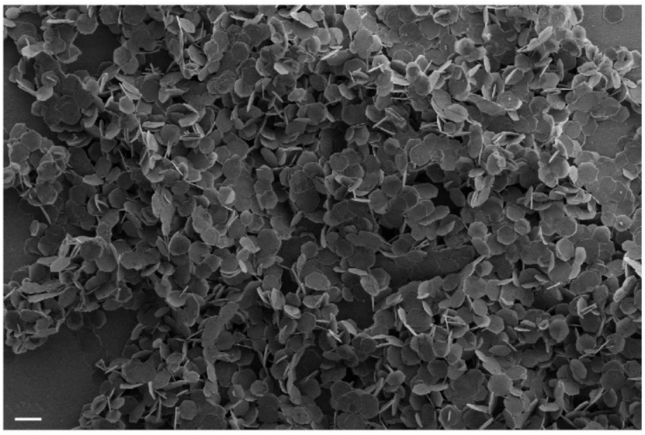

Supplementary Figure 1. SEM image of LDH-PEG nanosheets, scale bar: 1 μm. A representative image of three independent samples is shown.

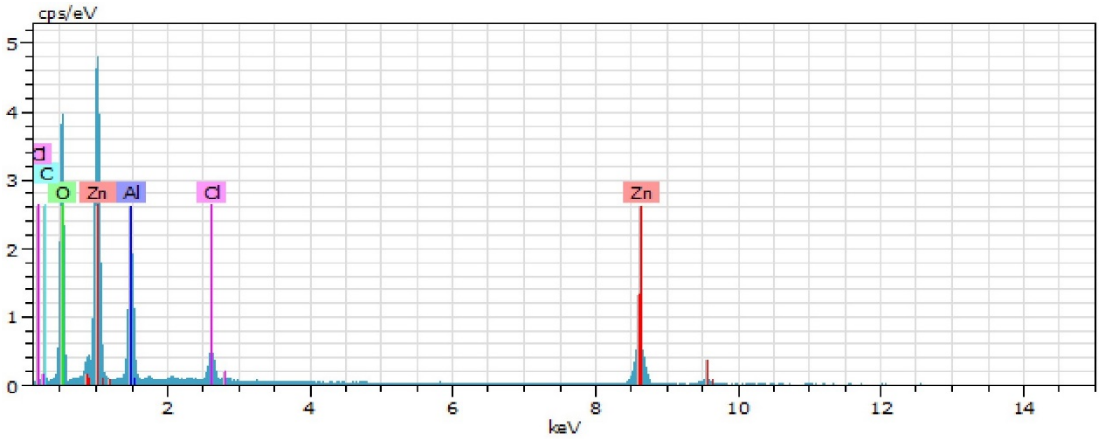

Supplementary Figure 2. EDS profile of LDH sample before PEGylation.

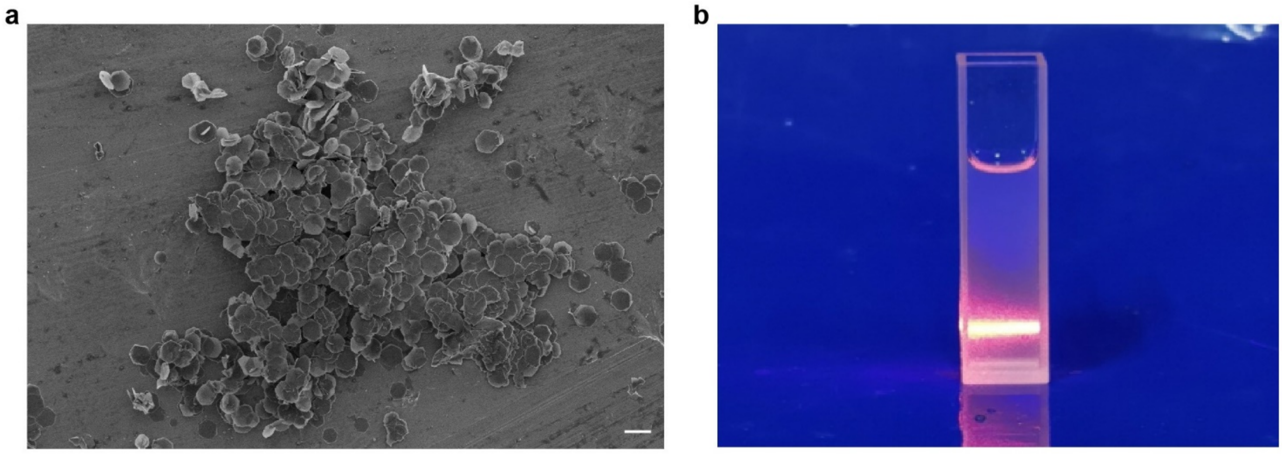

Supplementary Figure 3. (a) SEM image of LRT nanomedicines, scale bar: 1 μm. (b) Digital photo of LRT dispersed in PBS. A representative image of three independent samples is shown.

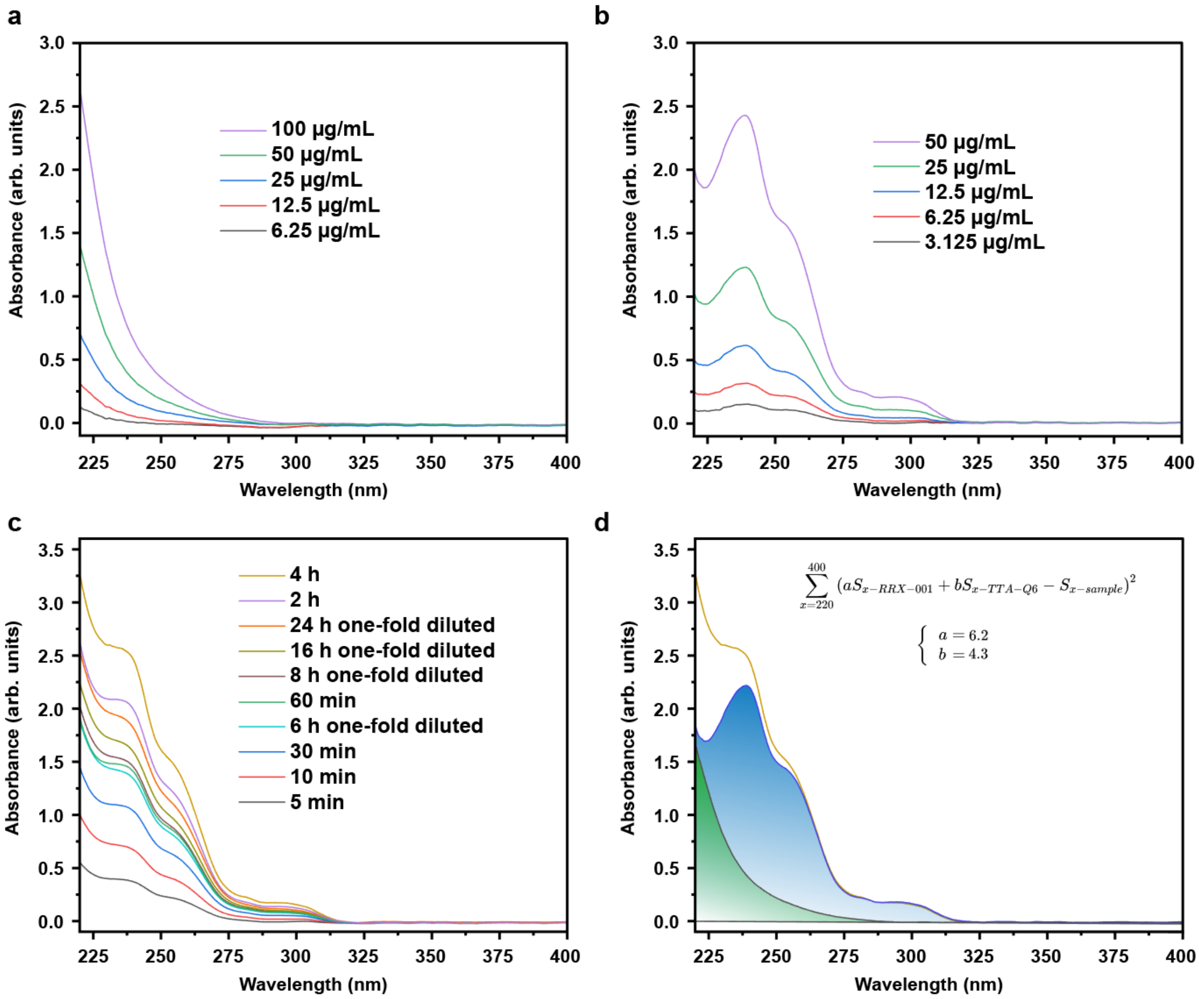

Supplementary Figure 4. UV absorbance of RRX-001 (a) and TTA-Q6 (b) standard solution at different concentrations. (c) UV absorbance of the supernatant after LRT released drugs in the phosphate buffer saline (pH 6.5) at different time. (d) Schematic illustration of the spectral separation by least-squares method. Representative of 3 independent experiments with similar results.

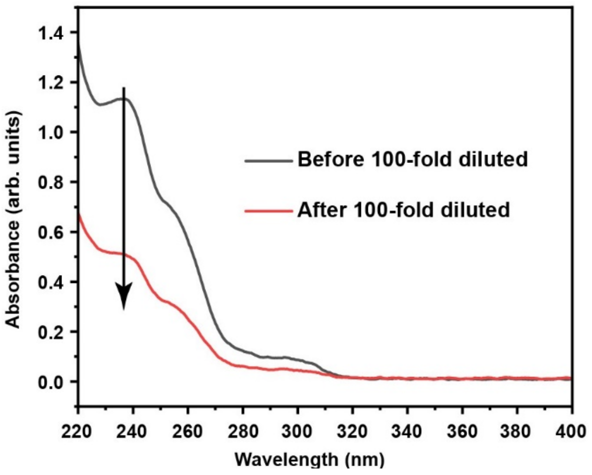

Supplementary Figure 5. UV absorbance of the supernatant before and after drug loading. Representative of 3 independent experiments with similar results.

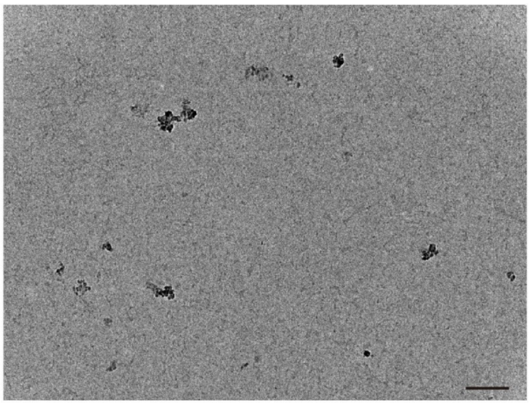

Supplementary Figure 6. TEM image of LRT sample after degradation for 72 h in PBS (pH = 6.5). Scale bar, 200 nm. A representative image of three independent samples is shown.

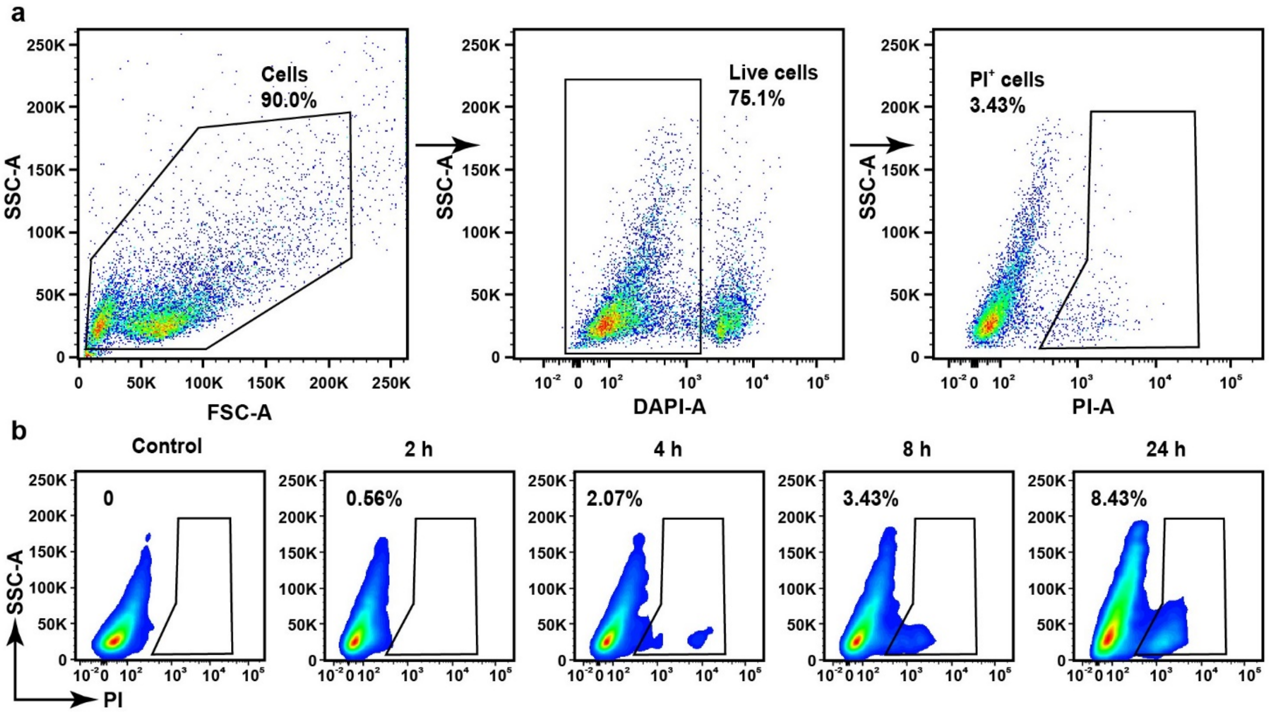

Supplementary Figure 7. (a) Gating strategy for flow cytometry LLC cells phagocytosis analysis. (b) Flow cytometry analysis of phagocytosis of LRT by LLC cells.

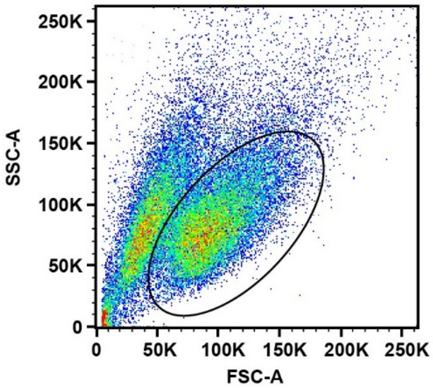

Supplementary Figure 8. Gating strategy for flow cytometric studies in Figure 3a.

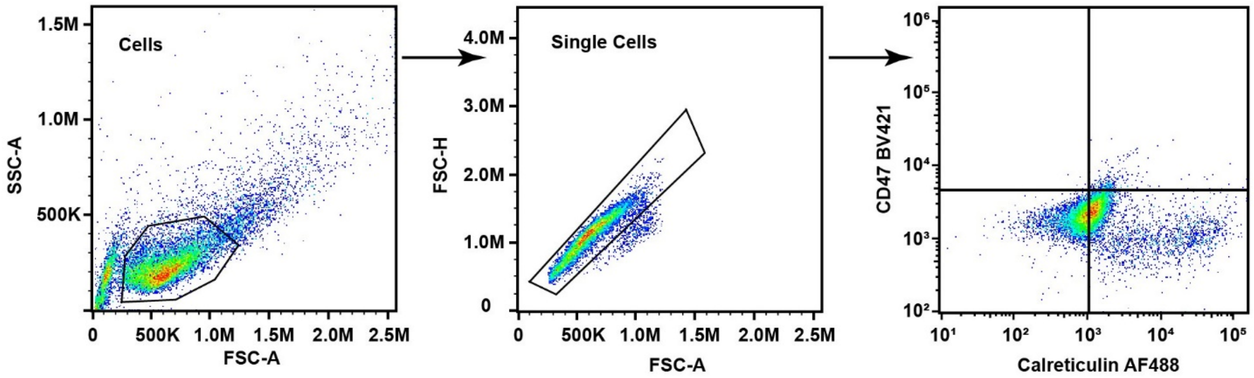

Supplementary Figure 9. Gating strategy for flow cytometric studies in Figure 3f.

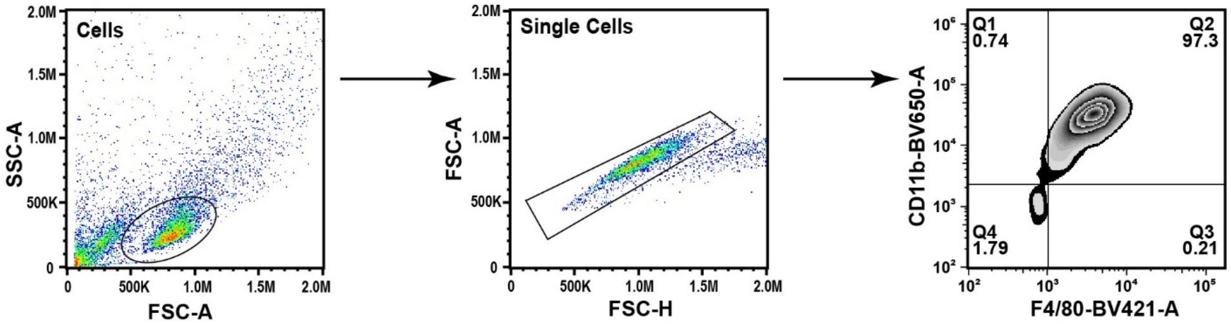

Supplementary Figure 10. Flow cytometry analysis for the isolated BMDMs.

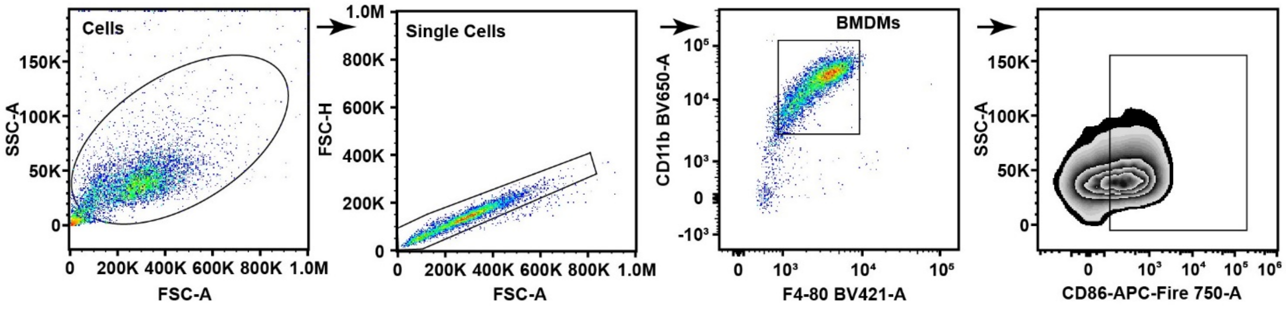

Supplementary Figure 11. Gating strategy for flow cytometric studies in Figure 4d.

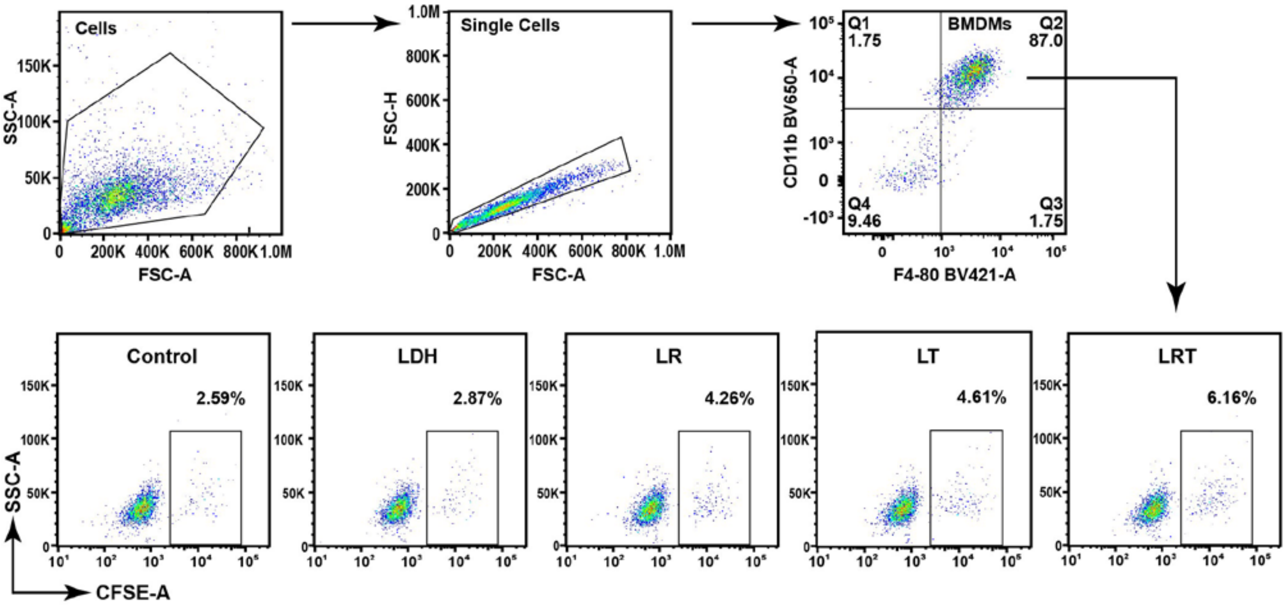

Supplementary Figure 12. Flow cytometry characterization of macrophage phagocytosis of cancer cells after different treatment and corresponding gating strategy.

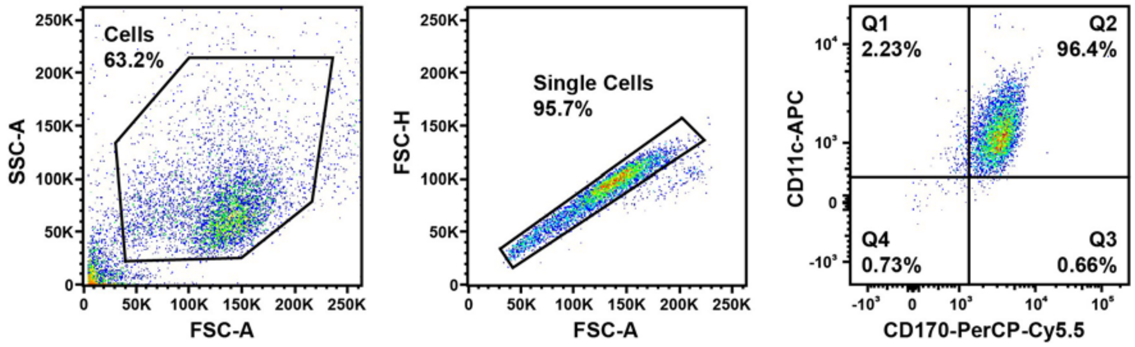

Supplementary Figure 13. Flow cytometry analysis for AMs purity.

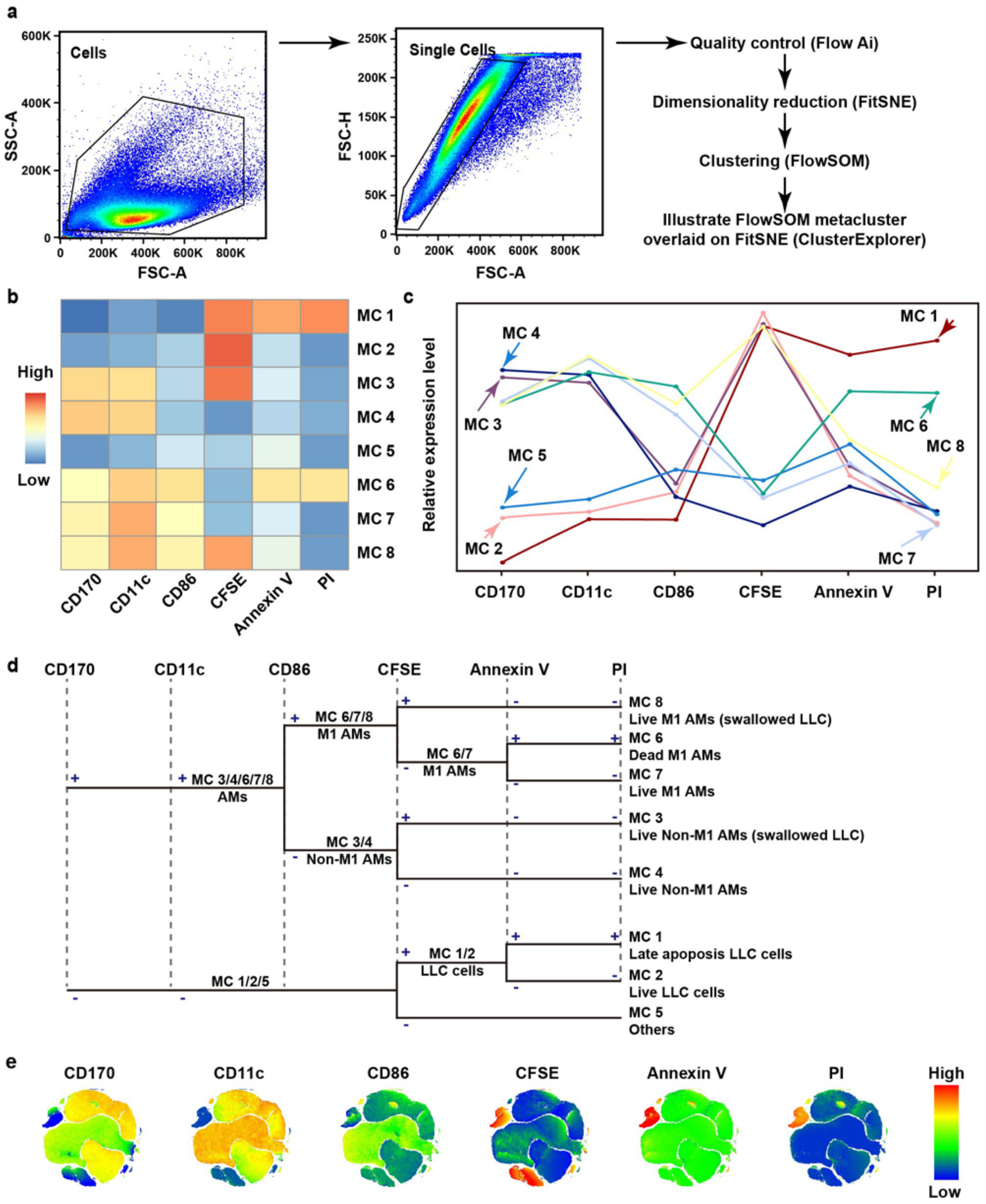

Supplementary Figure 14. (a) Analyzing strategy for flow cytometry analysis for the immune markers, tumor cell phagocytosis and killing effect of AMs. The relative expression of different cell marker in each FlowSOM metacluster (MC) showed in heatmap (b) and line graph (c). (d) The phenotype identity of each individual MC was defined by the expression of different cell marker. (e) Visualization of the phenotypic variation across all cell's subsets in FitSNE.

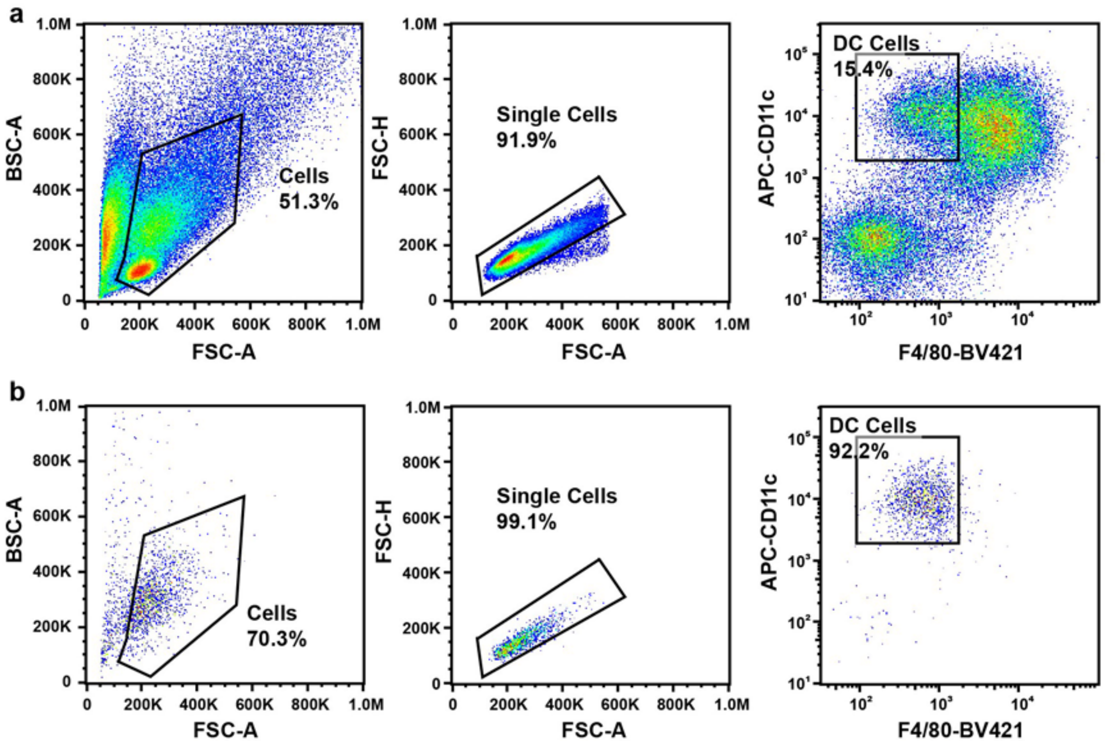

Supplementary Figure 15. (a) Gating strategy for sorting BMDCs. (b) BMDCs purity flow cytometric analysis.

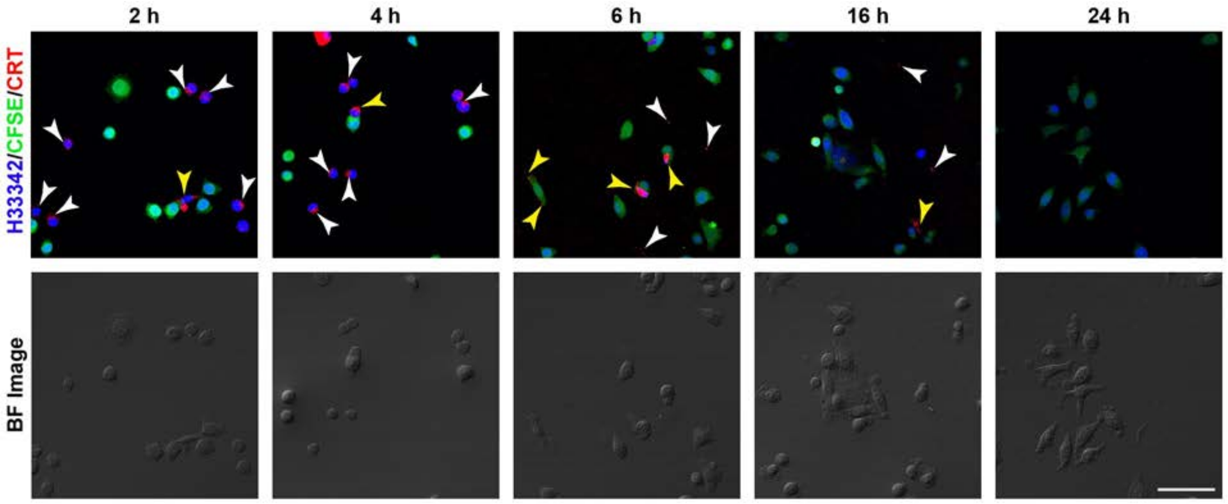

Supplementary Figure 16. Intracellular phagocytosis and processing of antigens in the LRT treated LLC (stained with CRT, red) cells by DC2.4 cells (stained with CFSE, green). The blue signals represent the cell nucleus. Scale bars, 80 μm. Yellow arrows represent cancer cells that have been phagocytosed by DC cells, and white arrows represent cancer cells that have not been phagocytosed. A representative image of three independent experiments is shown.

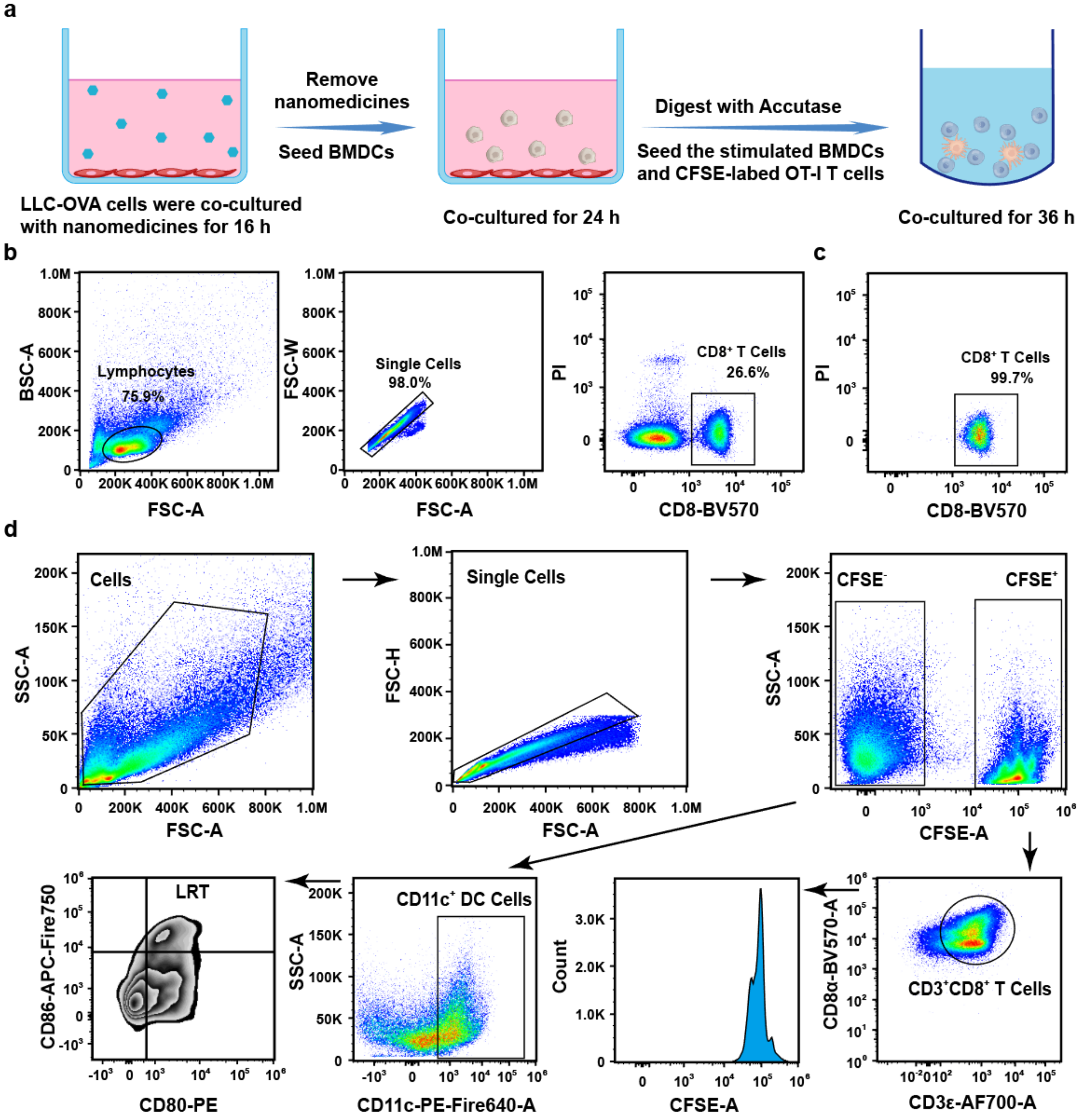

Supplementary Figure 17. (a) Schematic illustration of the flow cytometric phenotypic and T cells proliferation analysis. (b) Gating strategy for sorting $CD8^+$ OT-I T cells. (c) $CD8^+$ OT-I T cells purity flow cytometric analysis. (d) Gating strategy for DCs maturation and T cells proliferation flow cytometric analysis.

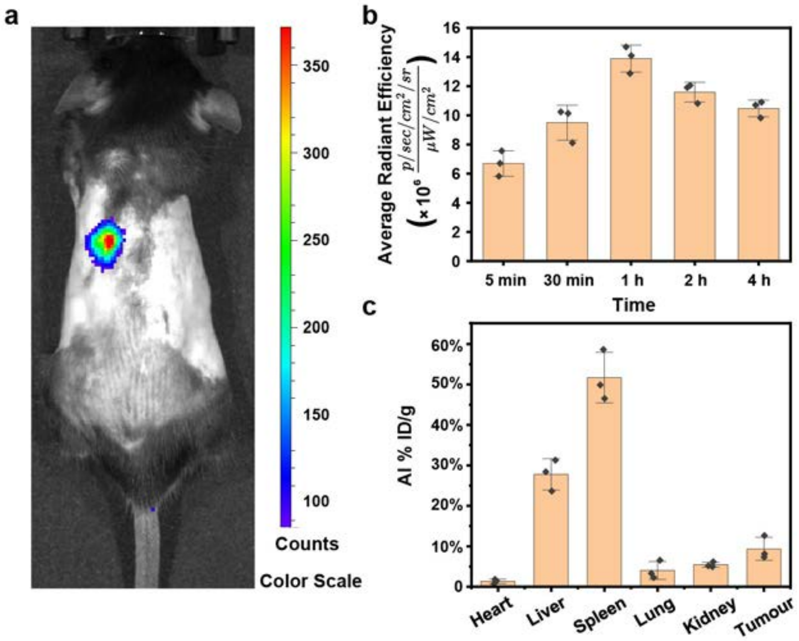

Supplementary Figure 18. (a) *In vivo* 2D bioluminescence of LLC-Luc cells. (b) Quantification of the fluorescence intensity at the left lung area of LLC tumor-bearing mice taken at preset time points post *i.v.* injection of LRT. (c) Distribution of Al element in main organs (hearts, livers, spleens, lungs, and kidneys) and tumors of mice at 1 h points after LRT administration. Data are expressed as mean ± SD (***N*** = 3 animals).

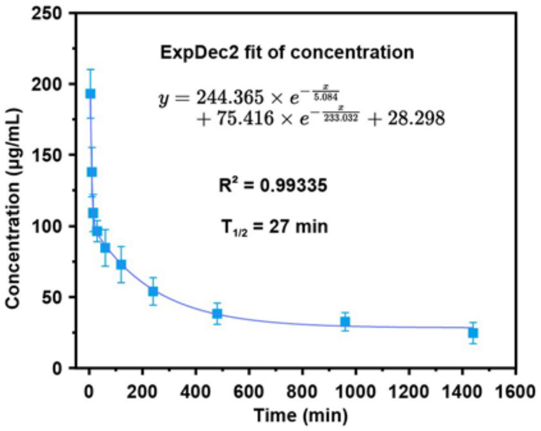

Supplementary Figure 19. Blood circulation curve of Al concentration after *i.v.* administration of LRT. The data were expressed as means ± SD (*N* = 3 animals).

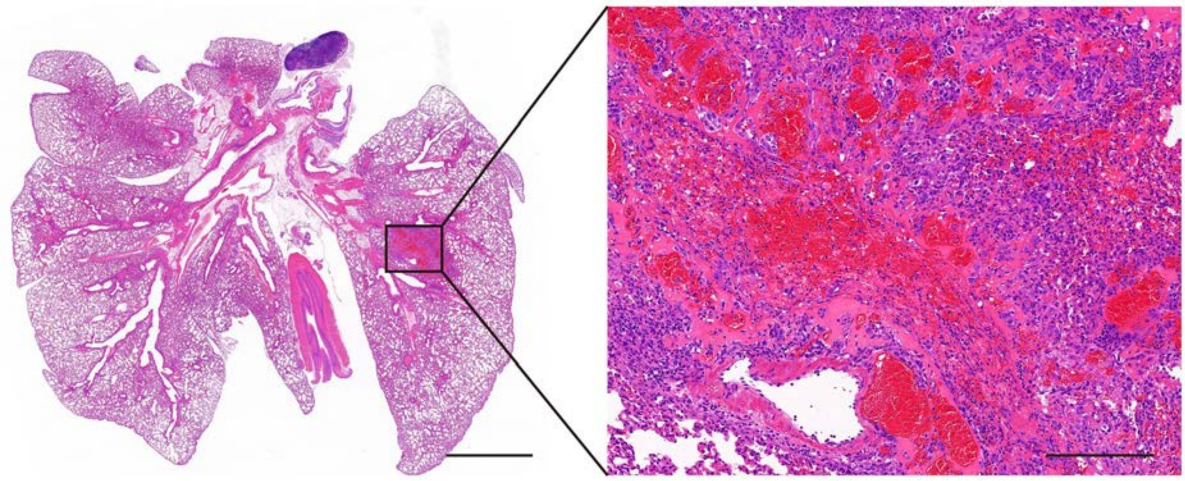

Supplementary Figure 20. H&E stained lung sections after LLC cells injection for 8 days. Scale bars: 2 mm, 200 μm corresponding. A representative image of three animals is shown.

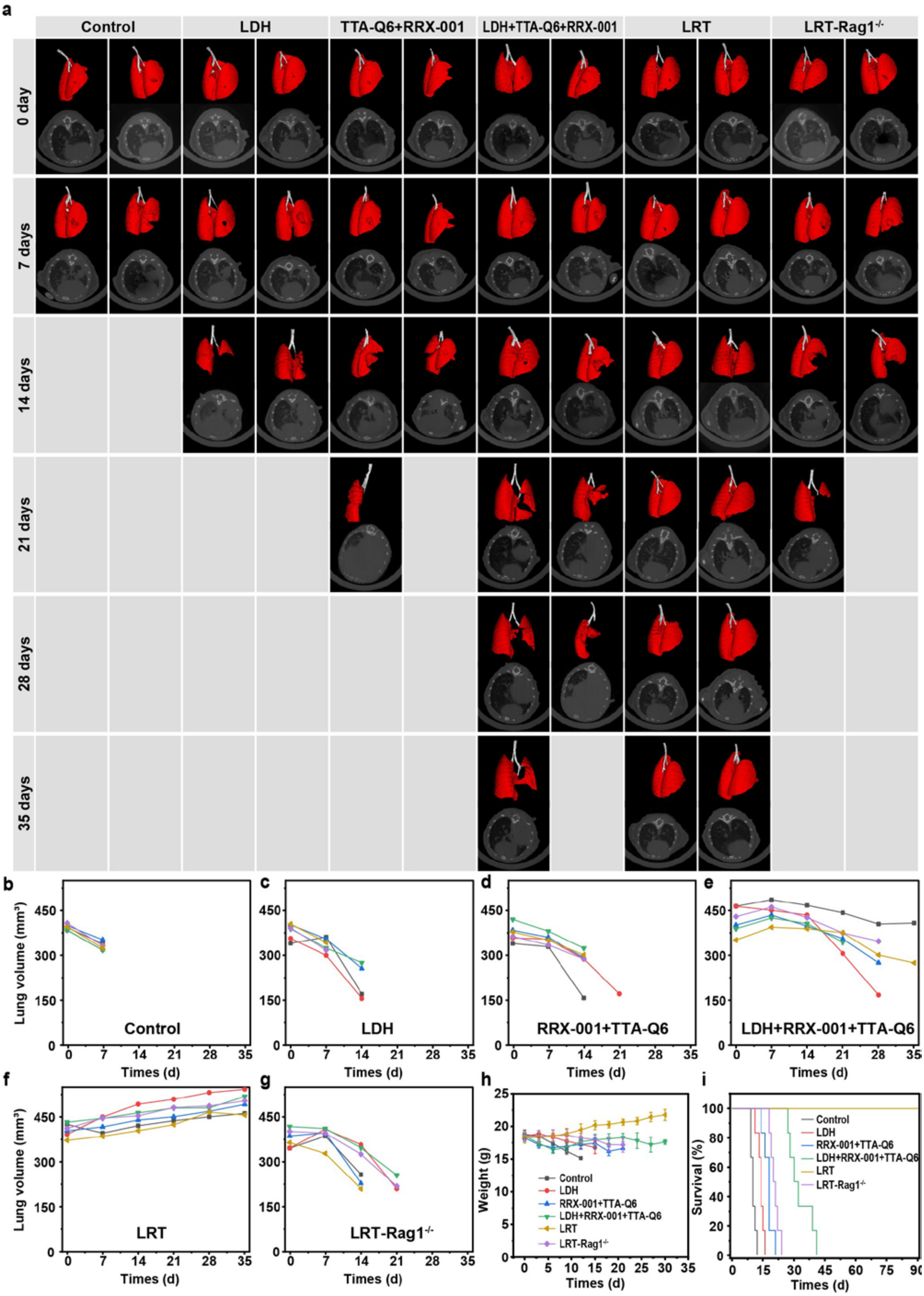

Supplementary Figure 21. *In vivo* immunotherapeutic outcomes by various treatments for orthotopic Lewis lung tumor model on C57BL/6J mice (6 animals per group). (a) Representative lung 3-dimensional reconstruction and corresponding CT transverse section

images from each group after varied treatments as indicated. (b-g) Quantifications of the healthy lung volumes by computational extraction through 3D reconstruction. The body weight fluctuation (h) and Kaplan-Meier survival curves (i) in between of LLC tumor-bearing mice after varied therapy. The data were expressed as means ± SD (N = 6 animals). The Rag1$^{-/-}$ groups were Rag1 deficient mice and treated with LRT, while other groups were wild-type C57BL/6J mice.

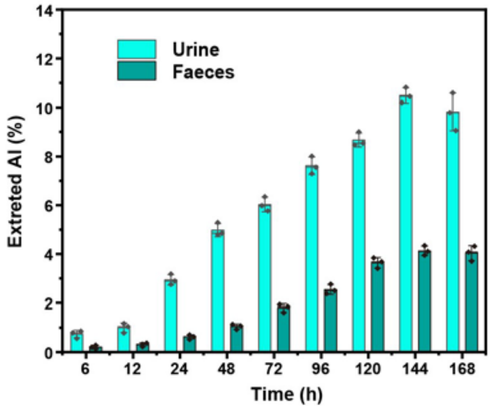

Supplementary Figure 22. Accumulated Al (in faeces and urine) excretion out of the mice body after the administration of LRT for different durations. The data were expressed as means ± SD (N = 3 animals).

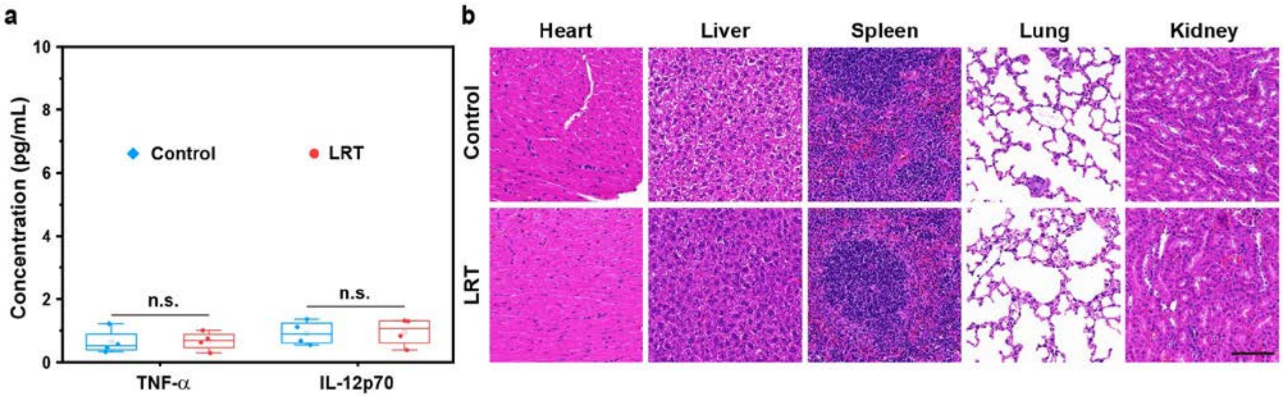

Supplementary Figure 23. Short-term *in vivo* safety evaluation of LRT nanomedicine. (a) TNF-α and IL12p70 levels in sera from the mice without tumors after LRT treatment. The LRT group were treated with LRT three times and the sera were harvested on day 8. N=4 animals. Statistical significances were calculated *via* Student's t test. Boxplots show the distribution of expression with the center of the box representing the mean, the center line correspond to the median, upper and lower bounds representing 75% and 25% percentiles, and upper and lower whiskers extending to the largest value no further than 1.5 times interquartile range from bounds of box. (b) H&E staining of major organs (heart, liver, spleen, lung, and kidney) harvested from the mice without tumor. The LRT group were treated with LRT three times and the organs were harvested on day 8. Scale bar, 100 μm. Representative of four animals from each experimental group.

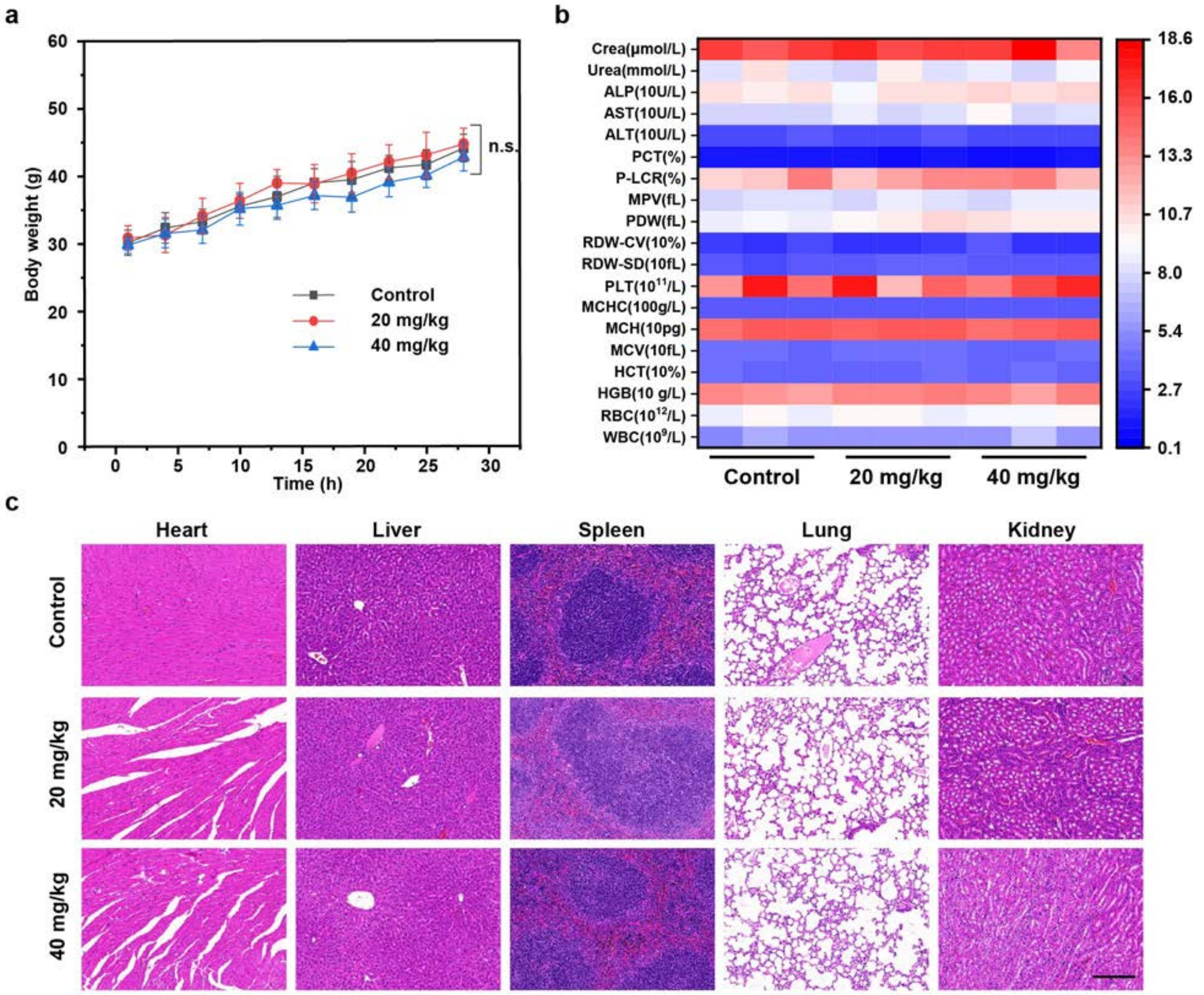

Supplementary Figure 24. Long-term *in vivo* safety evaluation of LRT nanomedicine. (a) Time-dependent body-weight curves of normal mice after treatment with PBS or PBS containing different doses of LRT nanosheets during an observation period of 28 days. The data were expressed as means ± SD ($N = 3$ animals). Statistical significances were calculated *via* Student's t test. n.s., not significant. (b) Hematological parameters of normal mice after various treatments on day 28. (c) H&E staining of major organs (heart, liver, spleen, lung, and kidney) harvested from normal mice after different treatments on day 28. Scale bar, 200 μm. Representative of three animals from each experimental group.

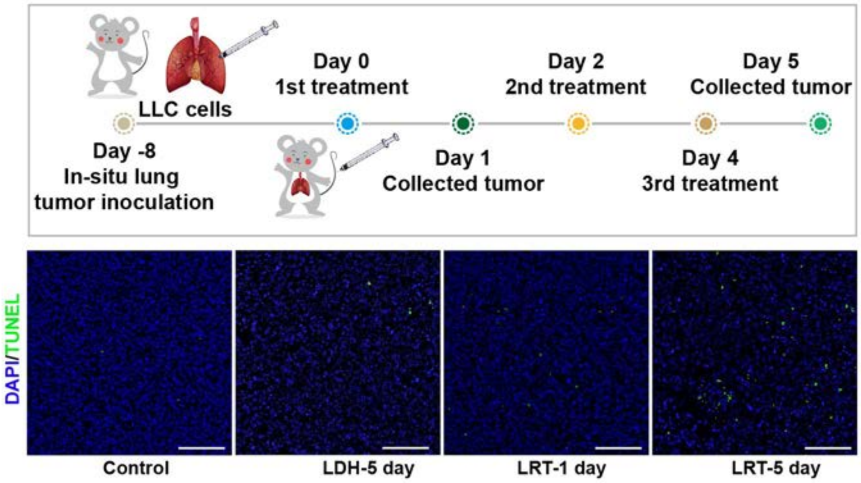

Supplementary Figure 25. Tunel staining of tumor sections after different treatment. Scale bars: 100 μm. Blue represent nucleus and green represents TUNEL signals. Representative of four animals from each experimental group.

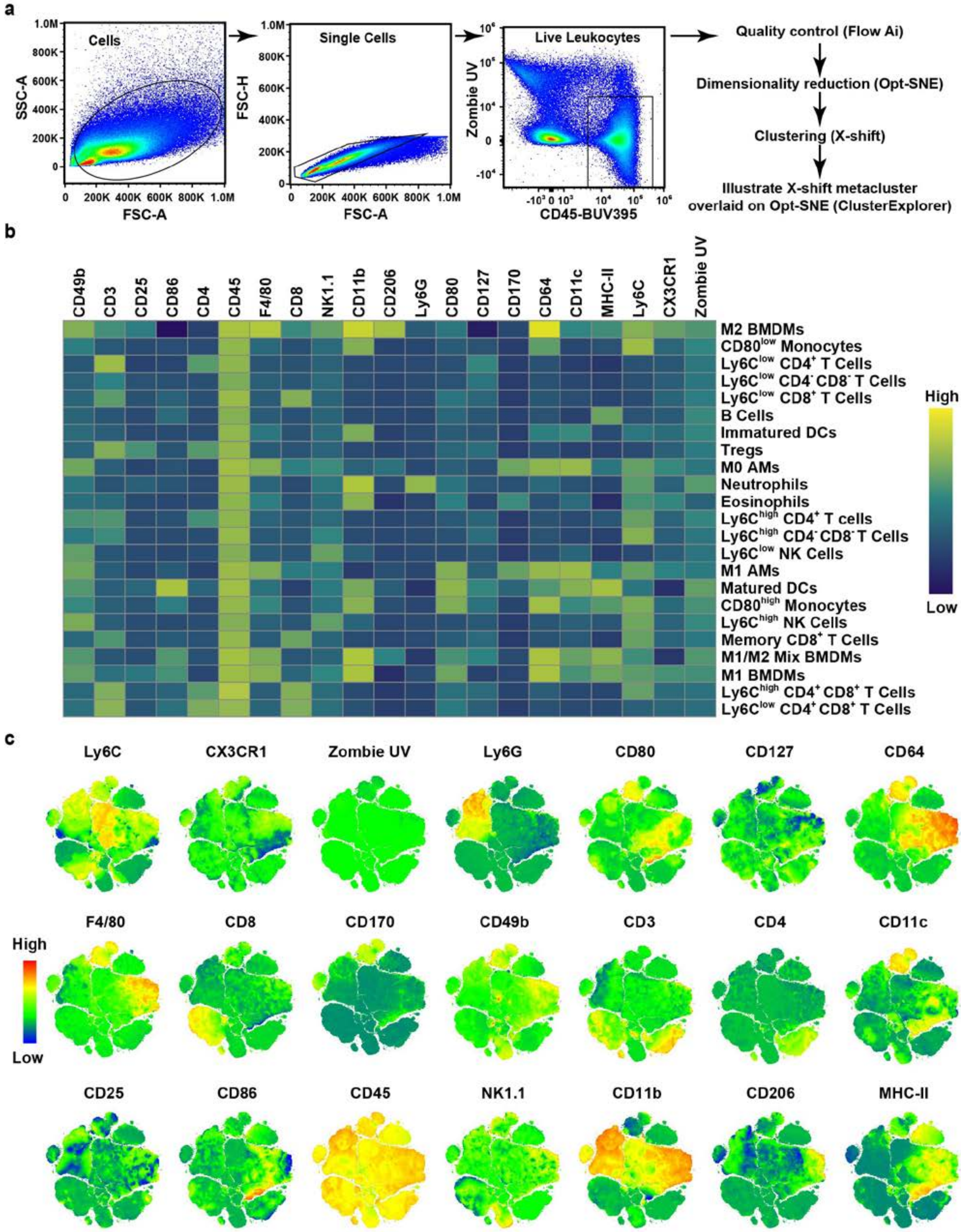

Supplementary Figure 26. (a) Analyzing strategy for flow cytometry analysis for the immune cells. Representative of 24 independent samples. (b) The relative expression of different cell marker in each x-shift cluster showed in heatmap. Merge results from 24 independent samples. (c) Visualization of the phenotypic variation across all cell's subsets in Opt-SNE. Merge results from 24 independent samples.

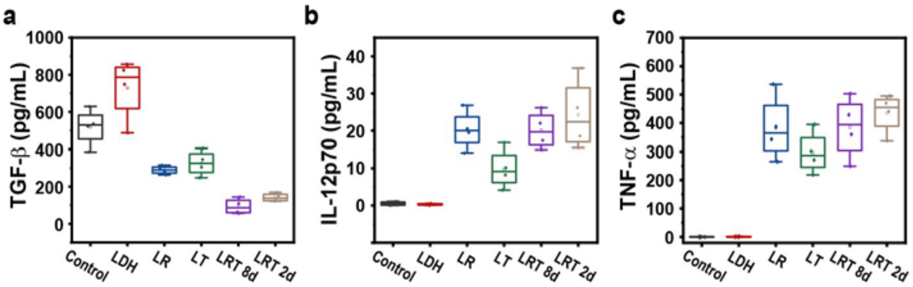

Supplementary Figure 27. TGF-β (a), IL12p70 (b) and TNF-α (c) levels in sera from each group after varied treatments as indicated. Boxplots show the distribution of expression with the center of the box representing the mean, the center line correspond to the median, upper and lower bounds representing 75% and 25% percentiles, and upper and lower whiskers extending to the largest value no further than 1.5 times interquartile range from bounds of box. *N*=4 animals.

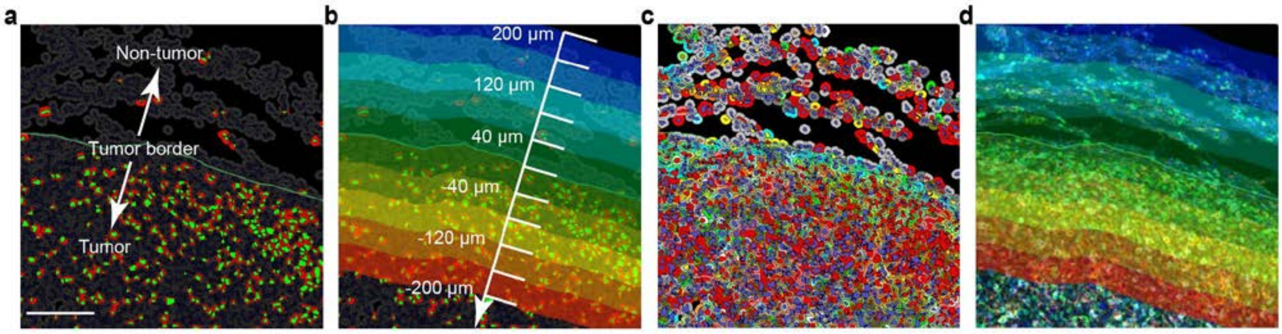

Supplementary Figure 28. (a) Images obtained after cell segmentation and cancer cell identification for mIHC image. The highlighted cells are cancer cells, and the gray cells are other cells. (b) Analysis area at different distances from the tumor border generated from infiltration analysis. (c) Images obtained after cell segmentation and identification for mIHC image. (d) The mIHC image and infiltration brand at different depth. Scale bar: 100 μm. Representative of 24 independent samples.

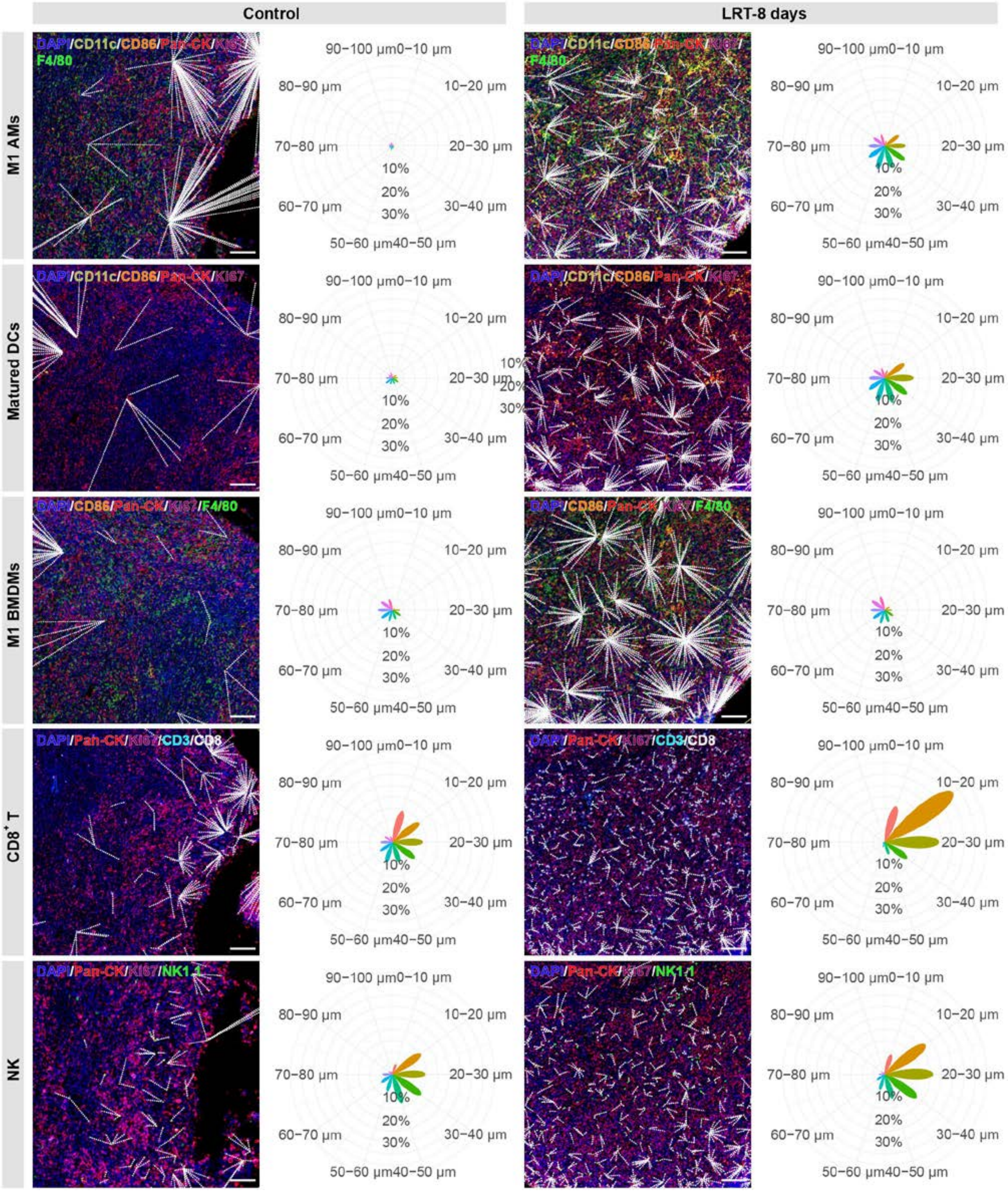

Supplementary Figure 29. Representative mIHC staining images of lung tumor in control and LRT-8 days groups, and corresponding spatial distribution of the nearest distance between cancer cells and immune cells (rose plot). The white dotted lines connect the cancer cell-immune cell pairs generated from the nearest neighbor analysis. The nearest neighbor analysis takes the cancer cell as the center and finds the closest immune cell to that cancer cell, so the result may be that one immune cell is the closest cell to multiple cancer cells. It also occurs that some immune cells are not selected as nearest cells by any other cancer cells. Scale bars, 100 μm. Representative of four animals from each experimental group.

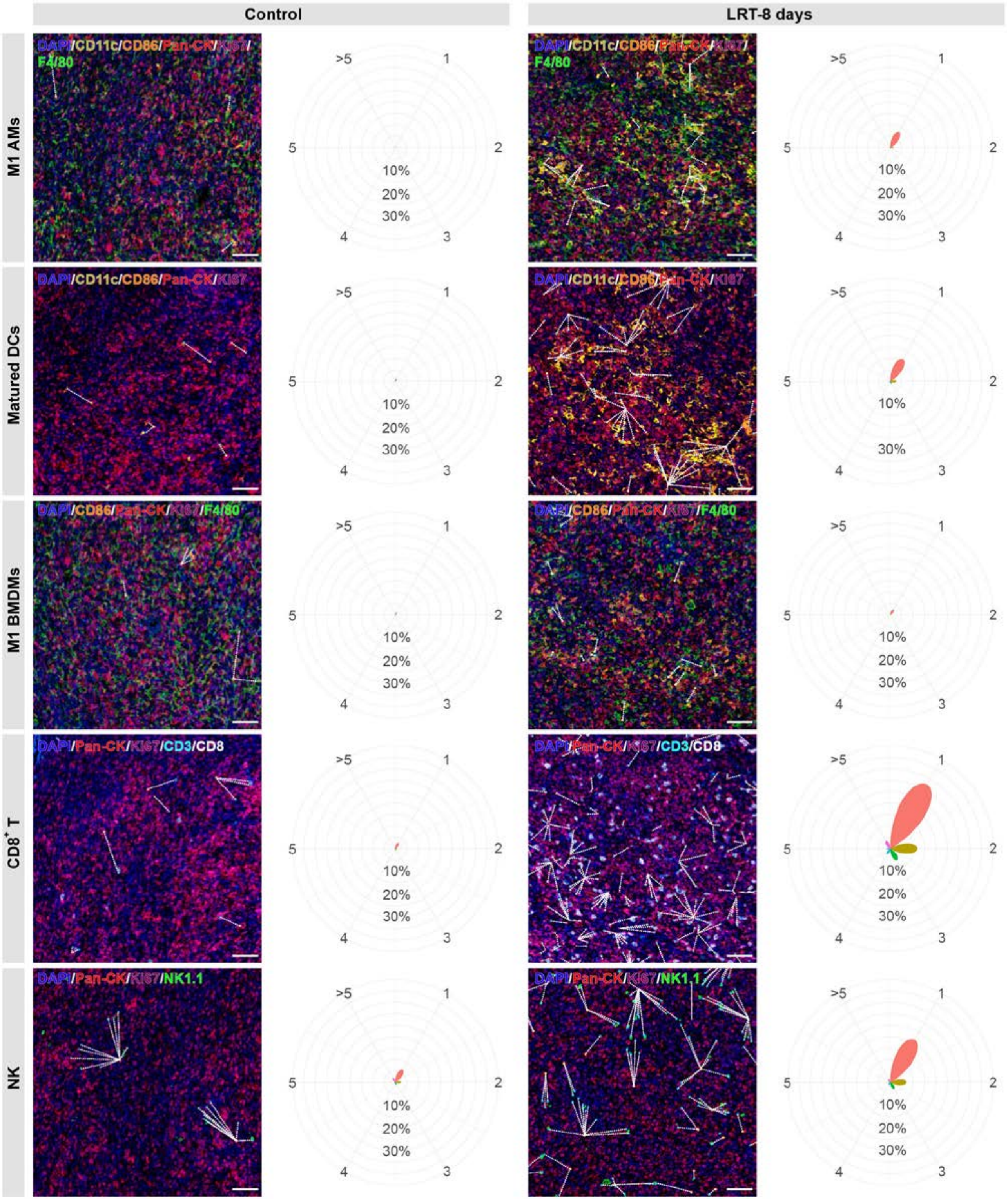

Supplementary Figure 30. Representative mIHC staining images of lung tumor in control and LRT-8 days groups, and corresponding quantitative data generated from proximity analysis (rose plot). Proximity analysis first matches immune cells to the nearest cancer cells to form cell pairs, and then analyzes the number of immune cells matched to that cancer cell within a radius of 100 μm with the cancer cell as the center, so the result may be that one cancer cell is the closest cancer cell to multiple immune cells. It also occurs that some cancer cells are not selected as nearest cells by any other immune cells. The white dotted lines connect the cancer cell-immune cell pairs generated from the proximity analysis. Scale bars, 100 μm. Representative of four animals from each experimental group.

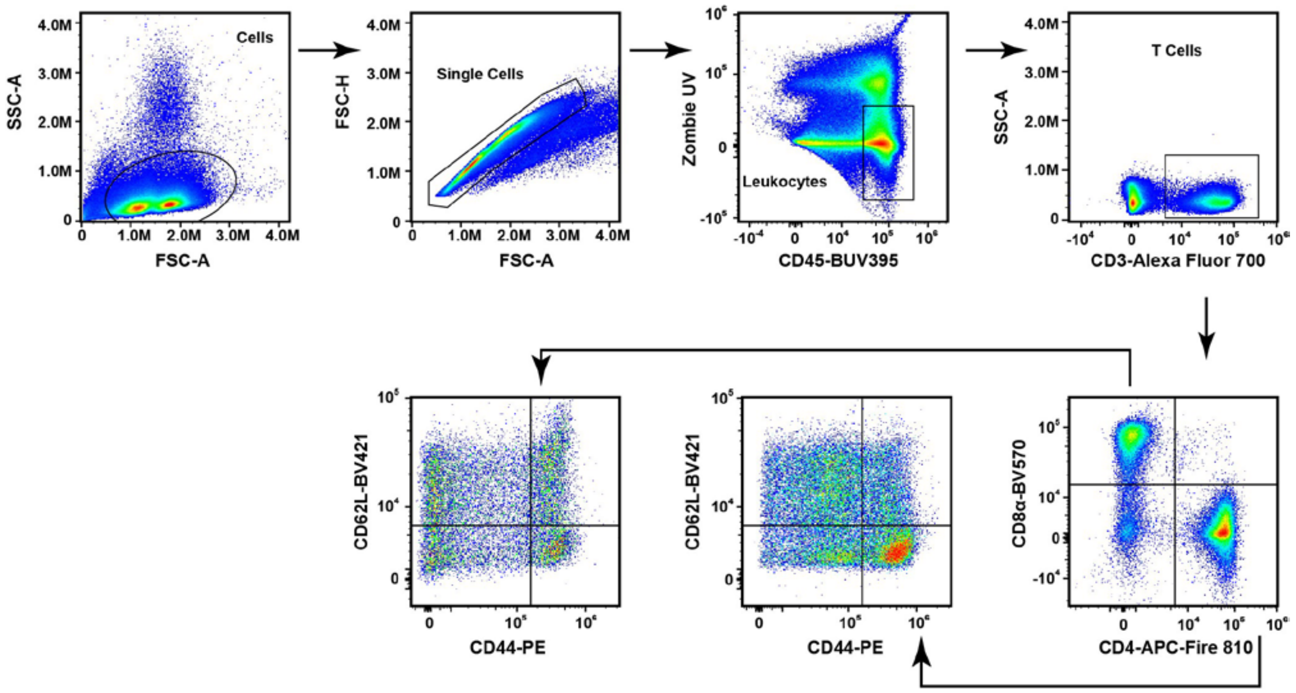

Supplementary Figure 31. Flow cytometry gating strategy for the analysis of memory T cells in the splenic lymphocytes.

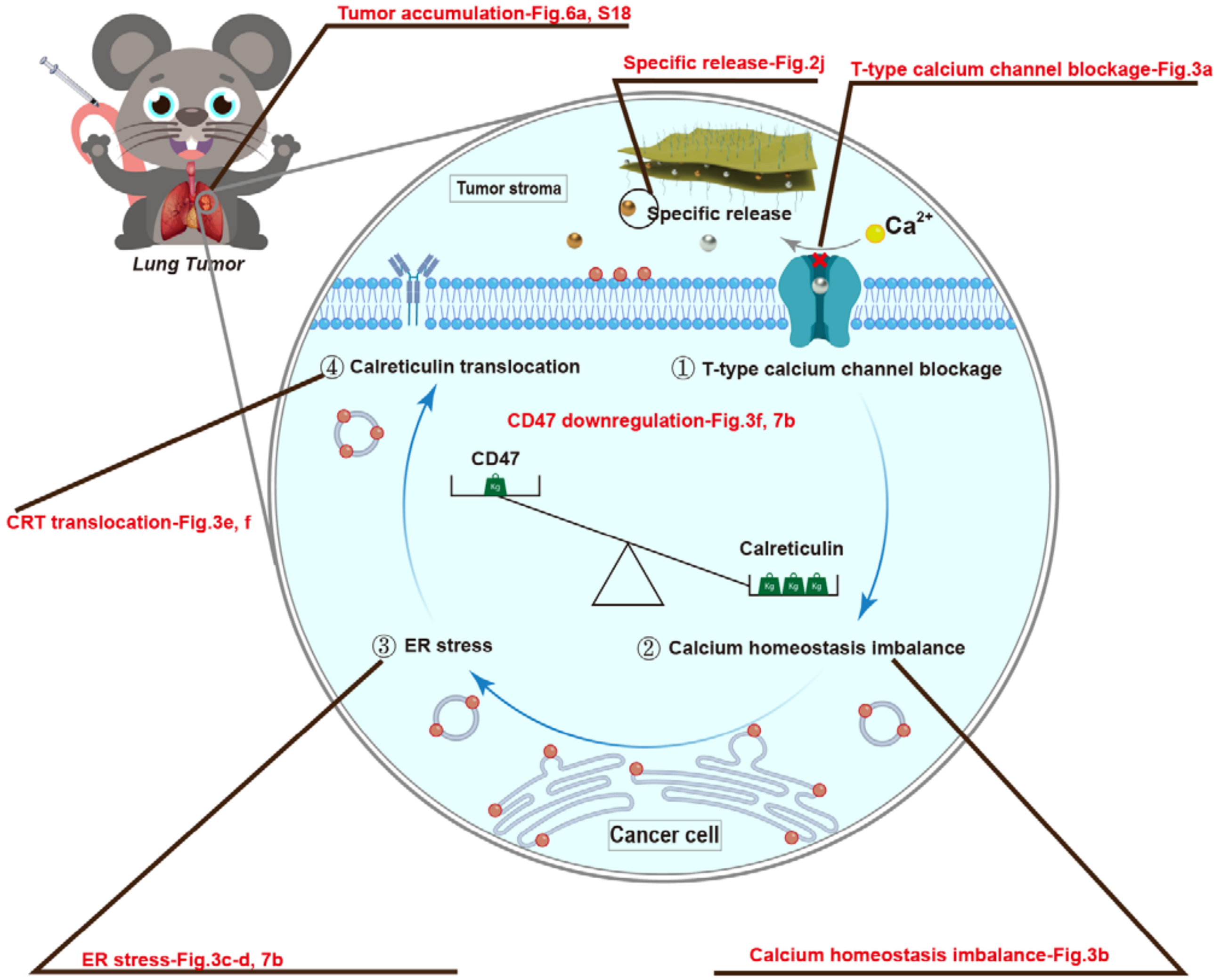

Supplementary Figure 32. Schematic diagram of the LRT therapy and relative evidence supporting each step: cancer cells part.

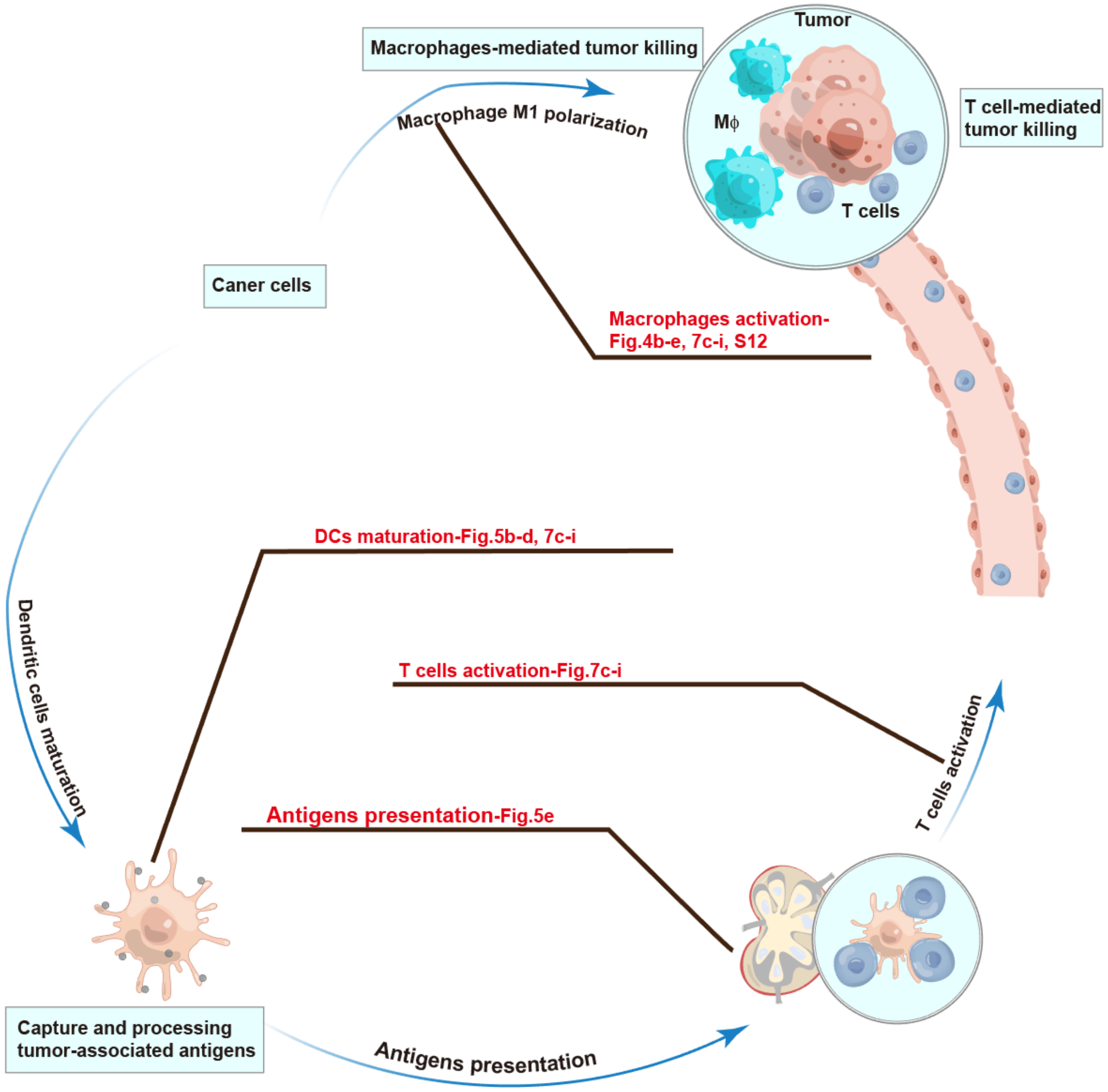

Supplementary Figure 33. Schematic diagram of the LRT therapy and relative evidence supporting each step: immune system part.
