# Supplementary material for: Nanomedicine-based co-delivery of a calcium channel inhibitor and a small molecule targeting CD47 for lung cancer immunotherapy
Source: Nat Commun. 2023 Nov 11;14:7306. doi: 10.1038/s41467-023-42972-2 (PMC10640620; doi:10.1038/s41467-023-42972-2)
Supplement: Supplementary file 3 — Reporting Summary [file 41467_2023_42972_MOESM3_ESM.pdf]

nature portfolio

Corresponding author(s): Ping Hu, Jianlin Shi

Last updated by author(s): Oct 19, 2023

Reporting Summary

Nature Portfolio wishes to improve the reproducibility of the work that we publish. This form provides structure for consistency and transparency in reporting. For further information on Nature Portfolio policies, see our Editorial Policies and the Editorial Policy Checklist.

Statistics

For all statistical analyses, confirm that the following items are present in the figure legend, table legend, main text, or Methods section.

n/a	Confirmed	
☐	☒	The exact sample size (*n*) for each experimental group/condition, given as a discrete number and unit of measurement
☐	☒	A statement on whether measurements were taken from distinct samples or whether the same sample was measured repeatedly
☐	☒	The statistical test(s) used AND whether they are one- or two-sided<br>*Only common tests should be described solely by name; describe more complex techniques in the Methods section.*
☐	☒	A description of all covariates tested
☐	☒	A description of any assumptions or corrections, such as tests of normality and adjustment for multiple comparisons
☐	☒	A full description of the statistical parameters including central tendency (e.g. means) or other basic estimates (e.g. regression coefficient) AND variation (e.g. standard deviation) or associated estimates of uncertainty (e.g. confidence intervals)
☐	☒	For null hypothesis testing, the test statistic (e.g. *F*, *t*, *r*) with confidence intervals, effect sizes, degrees of freedom and *P* value noted<br>*Give P values as exact values whenever suitable.*
☒	☐	For Bayesian analysis, information on the choice of priors and Markov chain Monte Carlo settings
☒	☐	For hierarchical and complex designs, identification of the appropriate level for tests and full reporting of outcomes
☒	☐	Estimates of effect sizes (e.g. Cohen's *d*, Pearson's *r*), indicating how they were calculated

Our web collection on statistics for biologists contains articles on many of the points above.

Software and code

Policy information about availability of computer code

Data collection	TEM (JEOL, JEM-2100F), SEM (Thermo Fisher, Quattro S), XRD (Rigaku, Ultima IV), FT-IR (Thermo Fisher, Nicolet iS 10), ICP-OES (Agilent, 715), Zeta potential (Malvern PANalytical, Zetasizer Nano ZS90), UV-Vis spectrum (Shimadzu, UV-3101PC), CLSM (Olympus FV1000), Flow cytometry (BD, Fortessa X20; Agilent, NovoCyte; SONY, ID7000; SONY, MA900), Western Blot (Bio-Rad, ChemiDoc XRS), In vivo imaging system (PerkinElmer, IVIS SpectrumCT), Micro-CT (PerkinElmer, Quantum CT; Bruker, SkyScan 1276CMOS), Elispot (Mabtech IRIS), Digital slide scanner (3DHISTECH, PANNORAMIC SCAN II; Akoya, PhenoImager Fusion)
Data analysis	All statistical analyses were performed on R 4.2.2 or Origin Pro (version 2021). All flowcytometry data were analyzed on Flowjo software package (TreeStar,version 10.8.1). Living imaging software (PerkinElmer, Living imaging version 4.5.2) was used to analyse bioluminescent and fluorescent images. All immunofluorescence images were guantified with HALO platform (version 3.4).

For manuscripts utilizing custom algorithms or software that are central to the research but not yet described in published literature, software must be made available to editors and reviewers. We strongly encourage code deposition in a community repository (e.g. GitHub). See the Nature Portfolio guidelines for submitting code & software for further information.

Data

Policy information about availability of data

All manuscripts must include a data availability statement. This statement should provide the following information, where applicable:

- Accession codes, unique identifiers, or web links for publicly available datasets
- A description of any restrictions on data availability
- For clinical datasets or third party data, please ensure that the statement adheres to our policy

The authors declare that all data needed to support the finding of this study are presented in the article, Source Data file and the Supplementary Information. A reporting summary for this article is available as a Supplementary Information file.

Research involving human participants, their data, or biological material

Policy information about studies with human participants or human data. See also policy information about sex, gender (identity/presentation), and sexual orientation and race, ethnicity and racism.

Reporting on sex and gender	N/A
Reporting on race, ethnicity, or other socially relevant groupings	N/A
Population characteristics	N/A
Recruitment	N/A
Ethics oversight	N/A

Note that full information on the approval of the study protocol must also be provided in the manuscript.

Field-specific reporting

Please select the one below that is the best fit for your research. If you are not sure, read the appropriate sections before making your selection.

☒ Life sciences ☐ Behavioural & social sciences ☐ Ecological, evolutionary & environmental sciences

For a reference copy of the document with all sections, see nature.com/documents/nr-reporting-summary-flat.pdf

Life sciences study design

All studies must disclose on these points even when the disclosure is negative.

Sample size	Sample sizes were determined based on estimates from pilot experiments, as statistical methods were not used for sample size calculation. For in vitro experiments, at least triplicates were included to enable statistical calculations. For in vivo studies, a sample size of 3-6 animals per treatment group was deemed sufficient to reliably detect statistically significant differences.
Data exclusions	No data were excluded from the analyses.
Replication	The experiments were replicated, and the findings were reproducible. Information on the experimental replicates can be found in the figure captions.
Randomization	Cells or mice were randomly assigned to different groups before treatment.
Blinding	The bio-TEM images were collected by experienced operators who were blinded to the treatment groups, and the investigators were blinded to group allocation during data analysis. For other experiments, the investigators maintained awareness of the experimental conditions throughout the data acquisition and analysis process, and the investigators were unbiased in both outcome assessment and conclusion drawing.

Reporting for specific materials, systems and methods

We require information from authors about some types of materials, experimental systems and methods used in many studies. Here, indicate whether each material, system or method listed is relevant to your study. If you are not sure if a list item applies to your research, read the appropriate section before selecting a response.

Materials & experimental systems

n/a	Involved in the study
☐	☒ Antibodies
☐	☒ Eukaryotic cell lines
☒	☐ Palaeontology and archaeology
☐	☒ Animals and other organisms
☒	☐ Clinical data
☒	☐ Dual use research of concern
☒	☐ Plants

Methods

n/a	Involved in the study
☒	☐ ChIP-seq
☐	☒ Flow cytometry
☒	☐ MRI-based neuroimaging

Antibodies

Antibodies used

The following primary antibodies were used for flow cytometry. They are listed as antigen first, following by linked Fluorescein, clone number, supplier, catalog number and the dilution ratio used, which were determined by antibody titration.

1) Anti-mouse CD47 BV421-linked, Clone: miap301, Biolegend, catalog number: 127527 (1:25 dilution);
2) Anti-mouse calreticulin AF488-linked, Clone: D3E6, CST, catalog number: 62304S (1:50 dilution);
3) Anti-mouse CD45 BUV395-linked, Clone: 30-F11, BD, catalog number: 564279 (1:320 dilution);
4) Anti-mouse F4/80 BV421-linked, Clone: BM8, Biolegend, catalog number: 123132 (1:160 dilution);
5) Anti-mouse SiglecF APC-linked, Clone: S17007L, Biolegend, catalog number: 155507 (1:80 dilution);
6) Anti-mouse SiglecF PE-Cy7-linked, Clone: S17007L, Biolegend, catalog number: 155528 (1:270 dilution);
7) Anti-mouse CD86 APC-Fire750-linked, Clone: GL-1, Biolegend, catalog number: 105046 (1:160 dilution);
8) Anti-mouse CD206 BV711-linked, Clone: C068C2, Biolegend, catalog number: 141727 (1:160 dilution);
9) Anti-mouse CD11c PE-Fire640-linked, Clone: QA18A72, Biolegend, catalog number: 161103 (1:330 dilution);
10) Anti-mouse CD11b BV650-linked, Clone: M1/70, Biolegend, catalog number: 101259 (1:270 dilution);
11) Anti-mouse Ly6G BV785-linked, Clone: 1A8, Biolegend, catalog number: 127645 (1:80 dilution);
12) Anti-mouse CD4 APC-Fire810-linked, Clone: GK1.5, Biolegend, catalog number:100479 (1:670 dilution);
13) Anti-mouse CD3ε AF700-linked, Clone: 500A2, Biolegend, catalog number:152316 (1:200 dilution);
14) Anti-mouse CD8α BV570-linked, Clone: 53-6.7, Biolegend, catalog number:100740 (1:160 dilution);
15) Anti-mouse CD127 PE-Cy5-linked, Clone: A7R34, Biolegend, catalog number:135015 (1:130 dilution);
16) Anti-mouse CD25 APC-linked, Clone: 3C7, Biolegend, catalog number:101909 (1:70 dilution);
17) Anti-mouse NK1.1 BV605-linked, Clone: PK136, Biolegend, catalog number:108739 (1:200 dilution);
18) Anti-mouse CD49b AF488-linked, Clone: DX5, Biolegend, catalog number:108913 (1:400 dilution);
19) Anti-mouse MHC-II PE-Fire810-linked, Clone: M5/114.15.2, Biolegend, catalog number:107667 (1:625 dilution);
20) Anti-mouse CX3CR1 Percp-Cy5.5-linked, Clone: SA011F11, Biolegend, catalog number:149009 (1:1250 dilution);
21) Anti-mouse Ly6C PB-linked, Clone: HK1.4, Biolegend, catalog number:128013 (1:1600 dilution);
22) Anti-mouse CD64 PE-Dazzle 594-linked, Clone: X54-5/7.1, Biolegend, catalog number:139319 (1:40 dilution);
23) Anti-mouse CD80 PE-linked, Clone: 16-10A1, Biolegend, catalog number:104707 (1:270 dilution);
24) Anti-mouse CD44 PE-linked, Clone: IM7, Biolegend, catalog number:103024 (1:20 dilution);
25) Anti-mouse CD62L BV421-linked, Clone: MEL-14, Biolegend, catalog number:104436 (1:160 dilution);
26) Anti-mouse CD16/32, Clone: S17011E, Biolegend, catalog number:156604 (1:200 dilution);

The following antibodies were used for immunofluorescence. They are listed as antigen first, following by clone number, supplier, catalog number and the dilution ratio used, which were determined by antibody titration.

1) Anti-mouse CD11c, Clone: D1V9Y, CST, catalog number:97585S (1:600 dilution);
2) Anti-mouse F4/80, Clone: D2S9R, CST, catalog number:70076S (1:450 dilution);
3) Anti-mouse CD86, Clone: E5W6H, CST, catalog number:19589S (1:300 dilution);
4) Anti-mouse CD3ε, Clone: E4T1B, CST, catalog number:78588S (1:400 dilution);
5) Anti-mouse CD8α, Clone: EPR21769, Abcam, catalog number:ab217344 (1:1500 dilution);
6) Anti-mouse pan-CK, Clone: C-11, Abcam, catalog number:ab7753 (1:300 dilution);
7) Anti-mouse Ly6G, Clone: E6Z1T, CST, catalog number:87048S (1:50 dilution);
8) Anti-mouse NK1.1, Clone: E6Y9G, CST, catalog number:39197S (1:300 dilution);
9) Anti-mouse Ki67, Clone: SP6, Abcam, catalog number:ab16667 (1:400 dilution);
10) Anti-mouse CD45, Clone: D3F8Q, CST, catalog number:70257S (1:200 dilution);
11) Anti-mouse CD31, Clone: D8V9E, CST, catalog number:77699S (1:200 dilution);
12) Anti-mouse CD47, Elabscience, catalog number:E-AB-40365 (1:50 dilution);
13) Anti-mouse CHOP, Elabscience, catalog number:E-AB-70087 (1:1200 dilution);
14) Anti-mouse IgG HRP-linked, ZSBIO, catalog number:PV-6002 (undiluted);
15) Anti-rabbit IgG HRP-linked, ZSBIO, catalog number:PV-6001 (undiluted);
16) Anti-mouse calreticulin, Clone: D3E6, CST, catlog number: 12238S (1:400 dilution);
17) Anti-rabbit IgG (H+L), $F(ab')_2$ Fragment Alexa Fluor® 647 linked, CST, catalog number: 4414S (1:1000 dilution).

The following primary antibodies were used for western blotting. They are listed as antigen first, following by clone number, supplier, and catalog number. All the antibodies were diluted and used following the supplier protocol.

1) Anti-mouse β-Actin, Clone: 8H10D10, CST, catalog number:3700S (1:1000 dilution);
2) Anti-mouse BIP, Clone: C50B12, CST, catalog number:3177T (1:1000 dilution);
3) Anti-mouse CHOP, Clone: L63F7, CST, catalog number:2895T (1:1000 dilution);
4) Anti-mouse IRE1α, Clone: 14C10, CST, catalog number:3294T (1:1000 dilution);

5) Anti-rabbit IgG HRP-linked, CST, catalog number:7074S (1:1000 dilution);
6) Anti-mouse IgG HRP-linked, CST, catalog number:7076S (1:1000 dilution).

Validation

All antibodies used in this manuscript were commercially available. The validation and quality control are performed by the corresponding vendors, and available on the manufactures' website and datasheet.

The following primary antibodies were used for flow cytometry.

1) Anti-mouse CD47 BV421
https://www.biolegend.com/en-us/products/brilliant-violet-421-anti-mouse-cd47-antibody-15866
2) Anti-mouse calreticulin AF488
https://www.cellsignal.cn/products/antibody-conjugates/calreticulin-d3e6-xp-rabbit-mab-alexa-fluor-488-conjugate/62304
3) Anti-mouse CD45 BUV395
https://www.bdbiosciences.com/en-us/products/reagents/flow-cytometry-reagents/research-reagents/single-color-antibodies-ruo/buv395-rat-anti-mouse-cd45.564279
4) Anti-mouse F4/80 BV421
https://www.biolegend.com/en-us/products/brilliant-violet-421-anti-mouse-f4-80-antibody-7199
5) Anti-mouse SiglecF APC
https://www.biolegend.com/en-us/products/apc-anti-mouse-cd170-siglec-f-antibody-16373
6) Anti-mouse SiglecF PE-Cy7
https://www.biolegend.com/en-us/products/pe-cyanine7-anti-mouse-cd170-siglec-f-antibody-20500
7) Anti-mouse CD86 APC-Fire750
https://www.biolegend.com/en-us/products/apc-fire-750-anti-mouse-cd86-antibody-13632
8) Anti-mouse CD206 BV711
https://www.biolegend.com/en-us/products/brilliant-violet-711-anti-mouse-cd206-mmr-antibody-12012
9) Anti-mouse CD11c PE-Fire640
https://www.biolegend.com/en-us/products/pe-fire-640-anti-mouse-cd11c-recombinant-antibody-21179
10) Anti-mouse CD11b BV650
https://www.biolegend.com/en-us/products/brilliant-violet-650-anti-mouse-human-cd11b-antibody-7638
11) Anti-mouse Ly6G BV785
https://www.biolegend.com/en-us/products/brilliant-violet-785-anti-mouse-ly-6g-antibody-12245
12) Anti-mouse CD4 APC-Fire810
https://www.biolegend.com/en-us/products/apc-fire-810-anti-mouse-cd4-antibody-19552
13) Anti-mouse CD3ε AF700
https://www.biolegend.com/en-us/products/alexa-fluor-700-anti-mouse-cd3epsilon-antibody-13779
14) Anti-mouse CD8α BV570
https://www.biolegend.com/en-us/products/brilliant-violet-570-anti-mouse-cd8a-antibody-7377
15) Anti-mouse CD127 PE-Cy5
https://www.biolegend.com/en-us/products/pe-cyanine5-anti-mouse-cd127-il-7ralpha-antibody-6193
16) Anti-mouse CD25 APC
https://www.biolegend.com/en-us/products/apc-anti-mouse-cd25-antibody-4512
17) Anti-mouse NK1.1 BV605
https://www.biolegend.com/en-us/products/brilliant-violet-605-anti-mouse-nk-1-1-antibody-8665
18) Anti-mouse CD49b AF488
https://www.biolegend.com/en-us/products/alexa-fluor-488-anti-mouse-cd49b-pan-nk-cells-antibody-2709
19) Anti-mouse MHC-II PE-Fire810
https://www.biolegend.com/en-us/products/pe-fire-810-anti-mouse-i-a-i-e-antibody-21099
20) Anti-mouse CX3CR1 Percp-Cy5.5
https://www.biolegend.com/en-us/products/percp-cyanine5-5-anti-mouse-cx3cr1-antibody-10461
21) Anti-mouse Ly6C PB
https://www.biolegend.com/en-us/products/pacific-blue-anti-mouse-ly-6c-antibody-6024
22) Anti-mouse CD64 PE-Dazzle 594
https://www.biolegend.com/en-us/products/pe-dazzle-594-anti-mouse-cd64-fcgammari-antibody-12424
23) Anti-mouse CD80 PE
https://www.biolegend.com/en-us/products/pe-anti-mouse-cd80-antibody-43
24) Anti-mouse CD44 PE
https://www.biolegend.com/en-us/products/pe-anti-mouse-human-cd44-antibody-2206
25) Anti-mouse CD62L BV421
https://www.biolegend.com/en-us/products/brilliant-violet-421-anti-mouse-cd62l-antibody-7164
26) Anti-mouse CD16/32
https://www.biolegend.com/en-us/products/trustain-fcx-plus-anti-mouse-cd16-32-antibody-17085

The following antibodies were used for immunofluorescence.

1) Anti-mouse CD11c
https://www.cellsignal.cn/products/primary-antibodies/cd11c-d1v9y-rabbit-mab/97585
2) Anti-mouse F4/80
https://www.cellsignal.cn/products/primary-antibodies/f4-80-d2s9r-xp-rabbit-mab/70076
3) Anti-mouse CD86
https://www.cellsignal.cn/products/primary-antibodies/cd86-e5w6h-rabbit-mab/19589
4) Anti-mouse CD3ε
https://www.cellsignal.cn/products/primary-antibodies/cd3e-e4t1b-xp-rabbit-mab/78588
5) Anti-mouse CD8α
https://www.abcam.com/products/primary-antibodies/cd8-alpha-antibody-epr21769-ab217344.html
6) Anti-mouse pan-CK
https://www.abcam.com/products/primary-antibodies/pan-cytokeratin-antibody-c-11-ab7753.html

7) Anti-mouse Ly6G
https://www.cellsignal.cn/products/primary-antibodies/ly-6g-e6z1t-rabbit-mab/87048
8) Anti-mouse NK1.1
https://www.cellsignal.cn/products/primary-antibodies/nk1-1-cd161-e6y9g-rabbit-mab/39197
9) Anti-mouse Ki67
https://www.abcam.com/products/primary-antibodies/ki67-antibody-sp6-ab16667.html
10) Anti-mouse CD45
https://www.cellsignal.cn/products/primary-antibodies/cd45-d3f8q-rabbit-mab/70257
11) Anti-mouse CD31
https://www.cellsignal.cn/products/primary-antibodies/cd31-pecam-1-d8v9e-xp-rabbit-mab/77699
12) Anti-mouse CD47
https://www.elabscience.com/p-cd47_polyclonal_antibody-318209.html
13) Anti-mouse CHOP
https://www.elabscience.com/p-chop_polyclonal_antibody-51669.html
14) Anti-mouse IgG HRP
http://www.zsbio.com/product/PV-6002
15) Anti-rabbit IgG HRP
http://www.zsbio.com/product/PV-6001
16) Anti-mouse calreticulin
https://www.cellsignal.cn/products/primary-antibodies/calreticulin-d3e6-xp-rabbit-mab/12238
17) Anti-rabbit IgG (H+L), $F(ab')_2$ Fragment Alexa Fluor® 647 linked
https://www.cellsignal.cn/products/secondary-antibodies/anti-rabbit-igg-h-l-f-ab-2-fragment-alexa-fluor-647-conjugate/4414

The following primary antibodies were used for western blotting.

1) Anti-mouse β-Actin
https://www.cellsignal.cn/products/primary-antibodies/b-actin-8h10d10-mouse-mab/3700
2) Anti-mouse BIP
https://www.cellsignal.cn/products/primary-antibodies/bip-c50b12-rabbit-mab/3177
3) Anti-mouse CHOP
https://www.cellsignal.cn/products/primary-antibodies/chop-l63f7-mouse-mab/2895
4) Anti-mouse IRE1α
https://www.cellsignal.cn/products/primary-antibodies/ire1a-14c10-rabbit-mab/3294
5) Anti-rabbit IgG HRP-linked
https://www.cellsignal.cn/products/secondary-antibodies/anti-rabbit-igg-hrp-linked-antibody/7074
6) Anti-mouse IgG HRP-linked
https://www.cellsignal.cn/products/secondary-antibodies/anti-mouse-igg-hrp-linked-antibody/7076

Eukaryotic cell lines

Policy information about cell lines and Sex and Gender in Research

Cell line source(s)	Mouse Lewis lung carcinoma cell line (LLC) were kindly provided by Cell Bank/Stem Cell Bank, Chinese Academy of Sciences. Luciferase-tagged LLC cell line was provided by iCell Bioscience Inc. DC2.4 mouse dendritic cell line was provided by YoBiBiotech Co., Ltd.
Authentication	Cell lines were authenticated by morphology, STR profiling, and PCR assays with species-specific primers.
Mycoplasma contamination	Cell lines tested negative for mycoplasma.
Commonly misidentified lines (See ICLAC register)	No commonly misidentified cell lines were used.

Animals and other research organisms

Policy information about studies involving animals; ARRIVE guidelines recommended for reporting animal research, and Sex and Gender in Research

Laboratory animals	6-week-old male C57BL/6J mice were purchased from Shanghai SLAC Laboratory Animal Co., Ltd. 6-month-old female OT-I mice were purchased from Shanghai Model Organisms Center, Inc. These mice were housed in ventilated stainless-steel cages under standard conditions (light: 12 h light/dark cycle, ambient temperature: 25 ± 2 °C, humidity: 60 ± 10%), which were fed with pellet food ad libitum and sterilized water.
Wild animals	This study did not involve wild animals.
Reporting on sex	Sex was not considered in this study design.
Field-collected samples	This study did not involve samples collected from the field.
Ethics oversight	All animal experiment procedures follow the guidelines of the Animal Care Ethics Commission of Shanghai Tenth People's Hospital, Tongji University School of Medicine (ID: SHDSYY-2018-Z0026, SHDSYY-2022-P0050 and SHDSYY-2023-Z0026-3)

Note that full information on the approval of the study protocol must also be provided in the manuscript.

Flow Cytometry

Plots

Confirm that:

☒ The axis labels state the marker and fluorochrome used (e.g. CD4-FITC).

☒ The axis scales are clearly visible. Include numbers along axes only for bottom left plot of group (a 'group' is an analysis of identical markers).

☒ All plots are contour plots with outliers or pseudocolor plots.

☒ A numerical value for number of cells or percentage (with statistics) is provided.

Methodology

Sample preparation	For the in vitro experiments, cells were dissociated into single-cell suspensions using Accutase and then stained with surface antigens. In some experiments, some cells were pre-stained with fluorescent dyes for cell labeling. For example, LLC cells were pre-stained with CFSE before co-culture with AMs in experiments investigating the polarization, killing effect, and swallowing function of AMs, while CD8-positive T cells were pre-stained with CFSE before co-culture with BMDCs in experiments investigating the maturation of BMDCs and the proliferation of T cells.<br><br>For the in vivo experiments, orthotopic lung tumors were harvested from mice in different groups, cut into small pieces, and digested using the Mouse Tumor Tissue Dissociation Kit, gentleMACS C tubes, and the gentleMACS™ Octo Dissociator. The resulting single-cell suspensions were filtered using 30 μm MACS SmartStrainers (Miltenyi Biotec), centrifuged, and resuspended in RBC lysis buffer (BD Biosciences). After being co-incubated for 5 minutes at room temperature, the single-cell suspensions were treated with an equal volume of PBS/10% FBS buffer and centrifuged, then washed with PBS buffer twice. The cells were then stained with live/dead dyes in PBS buffer, treated with anti-mouse CD16/32 to reduce nonspecific binding to FcRs, and finally stained with surface antigens.<br><br>Detailed experimental procedures can be found in the supplementary information.
Instrument	BD FortessaX20; Agilent NovoCyte; SONY ID7000; SONY MA900
Software	FlowJo software package (version 10.8.1)
Cell population abundance	BMDCs were sorted for >90% CD11c antigen positivity and F4/80 antigen negative, and CD8 positive T cells were sorted for >99% CD8 positivity and PI negative using a SONY MA900 sorter. Purity was determined by flow cytometry during and after the sort.
Gating strategy	In general, cells were first gated on FSC/SSC. Singlet cells were gated using FSC-H and FSC-A. Surface antigen gating was performed on the live cell population.

☒ Tick this box to confirm that a figure exemplifying the gating strategy is provided in the Supplementary Information.
